# Development of canine PD-1/PD-L1 specific monoclonal antibodies and amplification of canine T cell function

**DOI:** 10.1371/journal.pone.0235518

**Published:** 2020-07-02

**Authors:** Jin Wook Choi, Sita S. Withers, Hong Chang, Justin A. Spanier, Victoria L. De La Trinidad, Harmanpreet Panesar, Brian T. Fife, Roger Sciammas, Ellen E. Sparger, Peter F. Moore, Michael S. Kent, Robert B. Rebhun, Stephen J. McSorley

**Affiliations:** 1 Center for Immunology and Infectious Diseases, Department of Anatomy, Physiology and Cell Biology, School of Veterinary Medicine, University of California, Davis, California, United States of America; 2 Center for Companion Animal Health, Department of Surgical and Radiological Sciences, School of Veterinary Medicine, University of California, Davis, California, United States of America; 3 Center for Immunology, Department of Medicine, University of Minnesota Medical School, Minneapolis, Minnesota, United States of America; 4 Department of Medicine and Epidemiology, School of Veterinary Medicine, University of California, Davis, California, United States of America; 5 Department of Pathology, Microbiology and Immunology, School of Veterinary Medicine, University of California, Davis, California, United States of America; National Cancer Institute, UNITED STATES

## Abstract

Interruption of the programmed death 1 (PD-1) / programmed death ligand 1 (PD-L1) pathway is an established and effective therapeutic strategy in human oncology and holds promise for veterinary oncology. We report the generation and characterization of monoclonal antibodies specific for canine PD-1 and PD-L1. Antibodies were initially assessed for their capacity to block the binding of recombinant canine PD-1 to recombinant canine PD-L1 and then ranked based on efficiency of binding as judged by flow cytometry. Selected antibodies were capable of detecting PD-1 and PD-L1 on canine tissues by flow cytometry and Western blot. Anti-PD-L1 worked for immunocytochemistry and anti-PD-1 worked for immunohistochemistry on formalin-fixed paraffin embedded canine tissues, suggesting the usage of this antibody with archived tissues. Additionally, anti-PD-L1 (JC071) revealed significantly increased PD-L1 expression on canine monocytes after stimulation with peptidoglycan or lipopolysaccharide. Together, these antibodies display specificity for the natural canine ligand using a variety of potential diagnostic applications. Importantly, multiple PD-L1-specific antibodies amplified IFN-γ production in a canine peripheral blood mononuclear cells (PBMC) concanavlin A (Con A) stimulation assay, demonstrating functional activity.

## Introduction

Each year, 5,300 dogs per 100,000 are diagnosed with cancer, a rate that is approximately 10 times higher than the incidence in humans [1]. Despite the high incidence, treatment options have lagged behind human medicine, resulting in many dogs facing progressive disease with palliative care [2]. In contrast, over the last decade, several immunotherapies have been developed and approved for use in human cancers and have provided startling gains in survival for a cohort of patients who previously had few treatment options [3–6]. Similar immune-targeted approaches will likely be beneficial in canine cancer therapy, but few canine-specific immunological reagents have been generated for this purpose [7].

The most striking examples of effective human immunotherapies include CD19 specific chimeric antigen receptor (CAR)-T cell therapy and antibody-directed immune checkpoint blockade (ICB) approaches [8,9]. CD19 CAR-T cell therapy for relapsed or refractory CD19^+^ B-cell cancers now reports an 80% response rate [3]. However, this immune therapy can have side effects with grade 3 or 4 adverse events observed in 77% of treated patients [10]. Similarly, ICB therapy is now an established cancer immunotherapeutic approach that has proven to be highly effective in several human cancers [9]. This intervention primarily targets tumor specific CD8^+^ T cells with low to moderate anti-tumor activity due to cell surface immune checkpoint molecules that limit T cell responses. CTLA-4 is a classic example of an immune checkpoint molecule and is expressed on activated T cells following T cell Receptor (TCR) ligation [11]. Surface CTLA-4 binds to B7-1 and B7-2 with high affinity and competes with CD28, the activating ligand for B7-1/2 [11]. In a tumor setting, CTLA-4 expression by T cells can restrain an otherwise effective anti-tumor response [11]. Ipilimumab is a CTLA-4 blocking antibody that disengages this T cell inhibition and allows a host to eliminate cancerous cells more efficiently. Another well-known checkpoint interface is the interaction between PD-1 and the ligands, PD-L1 or PD-L2 [9,11,12]. PD-1 is expressed by activated T and B cells, as well as macrophages and NK cells, while PD-L1 can be displayed on a wide range of cells in nonlymphoid tissues, including tumor cells and tumor infiltrating immune cells [13–15]. PD-1 ligand expression in a tumor setting is often induced by inflammatory cytokines such as IFN-γ or TNF-α [11–13]. The consequence of PD-L1 or PD-L2 engagement in a tumor microenvironment is that PD-1 relays an inhibitory signal to infiltrating T cells via signaling through SHP2 [16,17].

Since the inhibitory effect of CTLA-4 occurs via competition for B7-1 and B7-2 expressed by lymph node antigen presenting cells, it is generally thought that this inhibition occurs during T-cell priming in lymphoid tissue. In contrast, PD-1 / PD-L1 triggered T-cell suppression was thought to occur mainly within the tumor environment where inflammatory cytokines are produced by local immune cells [9,11]. However, recent findings suggest that PD-1 mediated T-cell suppression can also occur during the T cell priming stage [11,18]. To liberate suppressed tumor-specific T-cells, numerous PD-1/PD-L1 blocking antibodies have been developed [19]. Pembrolizumab and Nivolumab were the first developed antibodies specific for human PD-1 and have proved to be effective in treating certain cancer types [9,20]. Atezolizumab, a human PD-L1-specific antibody approved to treat urothelial cancer and triple-negative breast cancer, is one of three commercially available PD-L1 monoclonal antibodies, along with Avelumab and Durvalumab [6,9,21–24]. Additional reagents have also been commercialized and a total of nine therapeutic monoclonal antibodies specific to PD-1 or PD-L1 are available in human medicine. These PD-1/PD-L1 reagents are currently being evaluated as a monotherapy or in combination with other anti-tumor approaches in 3000 clinical trials [19]. Thus, targeted interventions of the PD-1/PD-L1 interface has provided a highly effective strategy in a variety of human cancers.

Canine cancers bear many of the hallmarks of human cancers including tissue location, tumor progression, and response to chemotherapy and irradiation [7]. Thus, targeted immune interventions are likely to be an effective therapeutic approach for companion animal medicine [7]. However, canine lymphocyte populations are much less well defined and staining and blocking reagents that target specific immune checkpoint pathways are not yet widely available. CAR-T cell approaches for canine cancer are currently being explored and canine PD-1 and PD-L1 specific monoclonal antibodies have been described by several groups [7,25–28]. However, none of these approaches is commercially available and given the number of reagents used in human oncology, additional reagents will be needed for effective therapeutic strategies to be established. Here, we report the development of canine PD-1 and PD-L1 specific murine monoclonal antibodies that identify recombinant and native canine molecules in a variety of laboratory assays that are likely to have diagnostic relevance. In addition, these selected antibodies from our panel interrupt ligand binding and amplify canine T cell function *ex vivo*. Thus, these new reagents provide a foundation for exploration of PD-1 and PD-L1 therapy in canine oncology.

## Materials and methods

### Study animals

All animals were handled in accordance with institutional animal care and use committee (IACUC) practices at the University of California, Davis. Mouse use for this particular study was specifically approved by IACUC (20030). UC Davis animal care at facilities are accredited by the Association for the Assessment and Accreditation of Laboratory Animal Care (AAALAC). BALB/c mice (6–8 weeks of age) were purchased from The Jackson Laboratory (Bar Harbor, ME) for immunization experiments. Immunized mice were regularly monitored by campus veterinarian. Cervical dislocation was used for euthanasia of mice.

For *in vitro* assays to validate canine PD-1 and PD-L1 antibodies, peripheral blood mononuclear cells (PBMCs) were isolated from peripheral blood collected from healthy client-owned dogs (n = 7; ages 1.5–12 years) at the Center for Companion Animal Health (CCAH) (S1 Table). Canine donors were deemed healthy based on physical exam and normal blood profiles. Collection of peripheral blood samples from client-owned dogs was obtained with signed owner-informed consent, and was approved by both the UC Davis School of Veterinary Medicine Clinical Trials Review Board and the UC Davis IACUC (20375).

### Monoclonal antibodies and streptavidin

Alkaline phosphatase (AP)-conjugated streptavidin (Thermo Fisher Scientific, Waltham, MA) was used to detect biotinylated anti-mouse IgA (clone: 11-44-2; Thermo Fisher Scientific) and biotinylated PD-1Ig and PD-L1Ig in Western blot. R-Phycoerythrin (PE) conjugated Streptavidin (Prozyme, Hayward, CA) was used to generate tetramers. PE-conjugated anti-human IgG was used to aid B-cell enrichment. Horseradish peroxidase (HRP)-conjugated anti-mouse IgG (clone: Poly4053; Biolegend, San Diego, CA) was used for detection in initial binding enzyme-linked immunosorbent assay (ELISA) as well as PD-1/PD-L1 blocking ELISA. FITC-conjugated F(ab')2-Goat anti-Mouse IgG (H+L) (polyclonal, Thermo Fisher Scientific) was used as a secondary antibody to detect murine PD-1 and PD-L1 antibodies in fluorescence-activated cell sorting (FACS) staining of DH82 cells. Mouse IgG1 isotype control (clone: 11711; R&D Systems, Inc., Minneapolis, MN), IgG2a isotype control (clone: G155-788; BD Bioscience, San Jose, CA), and IgA isotype control (clone: S107; Thermo Fisher Scientific) antibodies were used as negative controls for FACS staining and functional assays. A secondary antibody for IgA isotypes, biotinylated anti-mouse IgA (Thermo Fisher Scientific) was used in combination with avidin-FITC (BD Bioscience, San Jose, CA) or streptavidin-APC (Thermo Fisher Scientific). More information on antibodies can be found in the supplementary data (S2 Table)

### Biochemical reagents and services

Q5 High-Fidelity DNA Polymerase (NEB, Ipswich, MA), NEB HiFi DNA Assembly Master Mix (NEB, Ipswich, MA), Zymoclean Gel DNA Recovery Kits (Zymo Research, Irvine, CA), PureLink Genomic DNA Mini Kit (Thermo Fisher Scientific), and Qiaprep Spin Miniprep Kit (Qiagen, Valencia, CA) were used for DNA manipulations. Genes were codon-optimized and synthesized at GenScript (Piscataway, NJ). Primers were synthesized at IDTDNA (Coralville, Iowa). Genes were sequenced at UCDNA Sequencing Facility (Davis, CA). Concanavalin A (ConA) (Sigma-Aldrich, St. Louis, MO) was used to stimulate PBMCs. Monophosphoryl Lipid A (MPLA) (Invivogen, San Diego, CA) and Complete Freund’s Adjuvant (CFA) (Sigma-Aldrich) were used as adjuvant to immunize mice. Ifn-γ (R&D Systems, Inc.) was used to stimulate DH82 cells. APC conjugation kit and PE conjugation kit (Abcam, Cambridge, MA) were used to label the anti-PD-1 and anti-PD-L1s. Zombie Aqua Fixable Viability Kit (Biolegend) was used to screen live and dead cells.

### DNA manipulation

The amino acid sequence of the extracellular domain of canine PD-1 (AB898677) and canine PD-L1 (AB898678) were fused with the Fc domain of human IgG1 to create recombinant canine PD-1Ig and PD-L1Ig [29]. *Drosophila melanogaster* signal sequence was placed at the N-terminus and biotin binding sequence was added at the C-terminus of both genes for biotinylation. PD-1Ig and PD-L1Ig genes were codon-optimized and synthesized by GenScript (Piscataway, NJ). BirA, an *Escherichia coli* (*E*. *coli*) biotin ligase, was PCR amplified from genomic DNA of *E*. *coli* (DH5α). PD-1Ig, PD-L1Ig, and BirA were cloned into pMT-BiP-V5-His-A (Life Technologies, Carlsbad, CA) creating pMT-PD-1Ig, pMT-PD-L1Ig, and pMT-BirA for expression under a metallothionein promoter. The full-length canine PD-1 and PD-L1 were also synthesized by GenScript with mouse PD-1 signal sequence at the N-terminus and cloned into pcDNA3.1 creating pcDNA3.1-PD-1 and pcDNA3.1-PD-L1. Unbiotinylated PD-1Ig (PD-1Ig-NB) and PD-L1Ig (PD-L1Ig-NB) were also prepared by not including biotin binding sequence, but, otherwise, with the same sequence as PD-1Ig and PD-L1Ig. PD-1Ig-NB and PD-L1Ig-NB were cloned into pMT-BiP-V5-His-A to create pMT-PD-1Ig-NB and pMT-PD-L1Ig-NB, respectively.

### Cell lines, cell culture and transfection

*D*. *melanogaster* S2 cells (K5130-01, September 2016) were purchased from Life Technologies (Carlsbad, CA). SP2/0 (CRL-1581, March 2017), CHO-K1(CCL-61, Aug 2017) and DH82 (CRL-10389, Aug 2017) cells were purchased from American Type Culture Collection (ATCC, Manassas, VA). Cell lines were maintained at minimum passage numbers to prevent any deviation of properties from the initial characterization. S2 cells were initially cultivated in Schneider’s Drosophila Medium (supplemented with 10% FBS and 0.5% Penicillin-Streptomycin). Transfection of S2 cells were performed using a commercial calcium phosphate transfection kit (Life Technologies, Carlsbad, CA). A stable cell line was generated through selection on blasticidin. The stable strain was cultivated in supplemented Schneider’s Drosophila Medium followed by Express Five SFM (supplemented with 0.5% Penicillin-Streptamycin) (Life Technologies) with shaking. Copper sulfate was added at 800 μM to induce the expression of the metallothionein promoter and biotin was added at 10 μg/ml for the biotinylation of PD-1Ig and PD-L1Ig. For culture of SP2/0 cells, hybridoma generation, and hybridoma culture, medium A from ClonaCell™-HY Hybridoma Kit (Stemcell Technologies Inc., Cambridge, MA) was used according to the manufacturer’s instructions. Hybridoma cells were also cultivated using DMEM complete medium (supplemented with 10% FBS, 50 μg/ml Gentamycin, 1% L-glutamine, 1% sodium pyruvate, 1% non-essential amino acids, 0.05mM 2-mercaptoethanol, and 1% 4-(2-hydroxyethyl)-1-piperazineethanesulfonic acid (HEPES)) or Hybridoma SFM (Life Technologies, Carlsbad, CA). CHO-K1 cells were transfected using JetPrime (Polyplus Transfection, New York, NY). CHO-K1 cells and their transfectants were cultivated in Ham’s F12K medium (supplemented with 10% FBS, 1% Penicillin-Streptomycin) (Life Technologies). DH82 cells were cultivated in DMEM complete medium (supplemented with 15% FBS, 1% Penicillin-Streptomycin, 1% L-glutamine, 1% sodium pyruvate, 1% non-essential amino acids, 0.05mM 2-mercaptoethanol, and 1% HEPES). DH82 was stimulated for 24 hours for upregulation of PD-L1 by supplementing with Ifn-γ at 10 ng/ml.

### Western blot of PD-1Ig and PD-L1Ig

After transfection of pMT-PD-1Ig and pMT-PD-L1Ig into S2 cells, culture supernatant was used to confirm correct expression of biotinylated PD-1Ig and PD-L1Ig on Western blot. Culture supernatant was run on a 4–15% TGX precast gels (Bio-Rad, Hercules, CA) and transferred to PVDF membrane. The transferred membrane was blocked with 5% skim milk, incubated with alkaline phosphatase conjugated streptavidin (SA-AP), and developed using NBT/BCIP (Thermo Fisher Scientific) as a substrate. PD-1Ig and PD-L1Ig were also used to test JC053 and JC071 in Western blot in combination with Goat anti-mouse IgG-AP conjugate (Bio-Rad, Hercules CA) as a secondary antibody.

### Protein purification and tetramer generation

PD-1Ig and PD-L1Ig were purified from the S2 cell culture supernatant by sample clarification via centrifugation and filtration followed by protein G agarose purification (Thermo Fisher Scientific) according to manufacturer’s protocol. PD-1Ig and PD-L1Ig were separately mixed with R-phycoerythrine (PE) conjugated streptavidin (SA-PE) in various ratios to determine the optimum ratio between Ig fused proteins and streptavidin. The correct ratio was confirmed on Western blot, which shows disappearing Ig-fusion protein band as all biotinylated Ig fusion proteins are associated with streptavidin. JC071, JC173, JC194, and JC205 were also purified using protein G agarose resin.

### Mouse immunization

Two immunization methods were employed to BALB/c mice for PD-1 and PD-L1 specific B-cell generation (IACUC 20030). In the first immunization method, 4 BALB/c mice were immunized with recombinant canine PD-1Ig or PD-L1Ig using an exponentially increasing dosing strategy with MPLA as adjuvant [30]. In this exponentially increasing dosing strategy (Exp-Inc), mice were given seven consecutive subcutaneous injections on the dorsal aspect of the neck every other day. Inoculum dose was increased two-fold at each injection. A total of 15 μg PD-1Ig or PD-L1Ig and 30 μg MPLA were delivered to each mouse. The same protocol was repeated for boosting immunizations to the same mice 28 days after the last priming dose. The second method used a single bolus subcutaneous injection with 50μg PD-1Ig or PD-L1Ig and 50ul Complete Freund’s Adjuvant (CFA) as an adjuvant diluted in PBS for priming. 28 days later, intravenous injection with 50μg PD-1Ig or PD-L1Ig in PBS was delivered to boost BALB/c mice in each group (n = 4). As a control, 2 mice, in each group, were given a subcutaneous injection with CFA and PBS for priming and intravenous injection with PBS only for the boost.

### Antigen specific B-cell enrichment and hybridoma generation

Mice were sacrificed by cervical dislocation, and lymph nodes and spleens were harvested three days after the last dose injection. Single cells were prepared and incubated with 24G2 Fc-receptor (Fc-R) blocking agent (Fc-R-block) (24G2 culture supernatant supplemented with 2% mouse serum and 2% rat serum), PE-conjugated PD-L1Ig tetramer (for anti-PD-1) or PD-1Ig tetramer (for anti-PD-L1), and PE-conjugated anti-human IgG1, sequentially. Cells were washed and incubated with anti-PE microbeads. Labelled cells were magnetically enriched via MACS (Miltenyi Biotech, Bergisch Gladbach, Germany) [31]. Hybridomas were generated using enriched cells according to the manufacturer’s protocol for the ClonaCell™-HY Hybridoma Kit (Stemcell Technologies Inc., Cambridge, MA). To summarize the procedure, enriched cells were fused with SP2/0 cells using 50% polyethylene glycol (PEG) solution. Fused cells were selected on semi-solid hypoxanthine-aminopterin-thymidine (HAT) medium. Selected colonies were maintained on 96-well plates until the initial ELISA screening for specificity.

### Hybridoma screening ELISA

Anti-PD-L1 hybridomas were initially screened by testing culture supernatant for antibody binding to PD-L1Ig on ELISA. 50ng of PD-L1Ig per well was coated on a High Bind 96-well plate at 4°C overnight. Plates were blocked in 5% BSA and incubated with hybridoma culture supernatants followed by HRP-conjugated anti-mouse IgG. 3,3',5,5'-Tetramethylbenzidine (TMB) was used as substrate and the reaction was stopped using sulfuric acid. Readings at 450 nm wavelength were used to measure the color change. As a decoy to screen out human IgG1 Fc-specific hybridomas, PD-1Ig was coated on a new set of ELISA plates and the same ELISA protocol was followed. Hybridomas that demonstrated positive signal on both PD-1Ig and PD-L1Ig were considered to be human IgG1 Fc-specific. Anti-PD-1 hybridomas were generated by the same protocol but a PD-L1Ig plate was used as a decoy. To test the PD-L1 antibodies’ ability to block interactions between PD-1 and PD-L1, unbiotinylated PD-L1Ig was coated on 96-well ELISA plates. Coated wells were blocked in 5% BSA and incubated with hybridoma supernatant, biotinylated PD-1Ig, and HRP-conjugated streptavidin (SA-HRP) sequentially. The PD-1 antibody blocking activity was also evaluated in the same way but using the opposite ligands. Again, TMB was used as a substrate and sulfuric acid was used to stop the reaction. Color change was measured at 450 nm wavelength.

### Canine PBMC isolation

Whole blood was obtained from healthy donor dogs for isolation of PBMCs to examine binding of PD-1 and PD-L1 antibodies to primary canine lymphocytes and monocytes after stimulation with various activation agents. Blood was processed for PBMCs by density centrifugation as previously described [32]. Briefly, whole blood was diluted in Hank’s Buffered Salt Solution (HBSS; Corning, Corning, NY, USA), layered over Histopaque 1077 (Sigma-Aldrich, Saint Louis, MO, USA), and centrifuged at 2000 rpm for 30 minutes at room temperature. The PBMC layer was extracted, washed with HBSS and then treated with RBC lysis buffer (420301, Biolegend, San Diego, CA, USA) for three minutes on ice for removal of contaminating red blood cells. PBMCs were washed again with HBSS, pelleted, and counted.

For a second set of experiments testing interferon-gamma (IFN-γ) production after PD-1/PD-L1 blockade, PBMCs were isolated from whole blood as previously described by Kol et al. [33] with the following modifications. Ficoll-Paque Plus (GE Healthcare, Piscataway, NJ) (9 ml) was diluted with 1.5 ml water to lower density to 1.066. Blood (10 ml) was diluted with 20 ml modified Tyrode’s buffer (12 mM NaHCO3, 138 mM NaCl, 2.9 mM KCl, 10 mM HEPES, and 1 mM EDTA). Histopaque 1119 (5 ml) was layered at the bottom of a 50 ml conical tube followed by 10 ml diluted Ficoll-Paque Plus, and a final top later of 30 ml diluted blood. The conical tube was centrifuged at 400g for 20 min without brake. The hazy interface layer between blood and Ficoll-Paque Plus was harvested, washed with PBS, and a final cell number was counted.

### Stimulation of PBMC subsets

Healthy canine donor PBMCs were stimulated with ConA 1 μg/ml for four days 37°C for testing of PD-1 expression by antibody JC053 using flow cytometry analysis. Unstimulated PBMC served as controls for resting cell expression of PD-1. For testing of the anti-PD-L1 JC071 antibody, PBMCs were stimulated with peptidoglycan (PGN, Sigma-Aldrich, Saint Louis, MO), or lipopolysaccharides (LPS, Sigma-Aldrich) for induction of PD-L1 expression on monocyte and dendritic cell subsets using a protocol previously described [34]. Briefly, PBMCs (10^6^ per ml) were treated with either PGN 1 μg/ml, LPS 10 ng/ml, or PBS (unstimulated control) in RPMI 1640 media (Gibco, Carlsbad, CA, USA) supplemented with 10% fetal bovine serum (FBS), 10 mM HEPES (Gibco), 100 U/ml penicillin and 100 μg/ml streptomycin (Gibco), 2 mM L-glutamine (Gibco), and 2-mercaptoethanol (Sigma-Aldrich) for three hours followed by a wash with media and resuspension in fresh media only, for the remaining incubation (21 hours) at 37°C. The next day cells were harvested and stained for flow cytometry analysis.

### Flow cytometry

For all experiments characterizing binding of PD-1 and PD-L1 monoclonal antibodies to cell lines, single cell suspensions were prepared in PBS and incubated with Zombie Aqua Fixable Viability Kit. Subsequently, cells were then incubated with Fc-R-block and appropriate antibodies sequentially. For mouse and CHO cells, 24G2 Fc-R-block was used. For dog PBMC, CLGL-90, and DH82, Fc Receptor Binding Inhibitor Antibody (Thermo Fisher Scientific) was used as Fc-R-block. Stained cells were fixed using 2% paraformaldehyde and stored at 4°C. All flow cytometry was performed on LSR Fortessa (BD Bioscience, San Jose, CA). FlowJo (version 10, BD Bioscience) software was used to analyze the data. CHO-PD1 was stained using Zombie Aqua Fixable Viability Kit, culture supernatant of JC053 hybridoma cells followed by biotinylated anti-mouse IgA and APC conjugated streptavidin. CLGL-90 was stained using JC053 culture supernatant, biotinylated anti-mouse IgA, and FITC-conjugated streptavidin. CHO-PDL1 was stained with Zombie Aqua Fixable Viability Kit and PE-conjugated PD-L1 antibodies. DH82 was stained with Zombie Aqua Fixable Viability Kit, purified PD-L1 antibodies, and FITC-conjugated F(ab')2-Goat anti-Mouse IgG (H+L).

For all experiments testing PD-1 or PD-L1 expression on PBMC subsets, single cell suspensions were initially stained with a fixable viability dye (LIVE/DEAD™ Fixable Near-IR Dead Cell Stain Kit, Invitrogen). For experiments testing ConA stimulation of PD-1 expression on T cell subsets within PBMCs, single cell suspensions were stained with cell surface markers including PacBlue-labeled anti-canine CD4 (clone YKIX302.9., Bio-Rad Laboratories, Hercules, CA), PerCP-EF 710-labeled anti-canine CD8 (YCATE55, Thermo Fisher Scientific) and APC-labeled JC053 (anti-canine PD-1). Cells were then fixed and permeabilized for intracellular staining using Fixation/Permeabilization concentrate and diluent (eBioscience, Carlsbad, CA, USA), and Permeabilization buffer (eBioscience) with FITC-labeled anti-human CD3 (clone CD3-12, Bio-Rad Laboratories) using conditions previously described [32]. For experiments testing PD-L1 expression on PBMC subsets after stimulation with either PBS, PGN, or LPS, single cell suspensions were first stained for viability and then treated with FcR blocking antibody (Thermo Fisher Scientific) 4C for 20 min. Cells were next stained for cell surface markers including PerCP-eFluor 710-labeled anti-canine CD5 (clone YKIX322.3, Thermo Fisher Scientific), FITC-labeled anti-canine MHCII (clone YKIX334.2, Bio-Rad Laboratories), Qdot 605-conjugated anti-human CD14 (clone Tuk4, Thermo Fisher Scientific), PE-labeled anti-canine CD11c (clone CA11.6A1, Laboratory of Peter Moore, University of California Davis), and APC-labeled JC071 (anti-PD-L1). Gating was based on isotype controls or biological controls that included stimulated and unstimulated cells.

Stained PBMC were washed in buffers and under conditions as previously described [32]. Cells were suspended in 1% paraformaldehyde (Affymetrix) and stored at 4°C for subsequent acquisition on a Becton Dickenson LSRII flow cytometer. A minimum of 500,000 events were collected. Compensation settings were conducted using BD CompBeads (BD Biosciences) and flow cytometry data analyzed with FlowJo software (Version 10, BD).

### IFN-γ assay

0.5 million canine PBMCs were stimulated with 1 μg/ml ConA and cultivated in 200ul DMEM complete medium in a U-bottom 96-well plate for 96 hours with or without PD-1 or PD-L1 antibodies. After cultivation, supernatant was collected and IFN-γ production was evaluated using an IFN-γ Quantikine ELISA Kit (R&D Systems, Inc., Minneapolis, MN).

### Immunohistochemistry

CHO-K1 and CHO-PD1 cells were fixed with 10% neutral buffered formalin, prepared in Histogel (Thermo Fisher Scientific, Waltham, MA), and embedded in paraffin. Paraffin blocks of cells or tissues were cut at 4um and deparaffinized in Xylene and antigens were retrieved in citrate buffer at pH 6. Samples were blocked with normal horse serum and incubated with JC053 or IgA isotype control as primary antibody. Biotinylated anti-mouse IgA (Thermo Fisher Scientific) was used as the secondary antibody. VECTASTAIN Elite ABC HRP Kit and DAB Peroxidase (HRP) Substrate Kit (Vector Laboratories, Burlingame, CA) were subsequently used for visualization.

### Statistics

Comparisons of frequencies PD-1^+^ and PD-L1^+^ cells within different PBMC subsets before and after stimulation were performed using a nonparametric 2-tailed Mann-Whitney test and GraphPad Prism (GraphPad Software, San Diego CA). For analysis of IFN-γ assay, ordinary one-way ANOVA with GraphPad Prism (GraphPad Software) was used. P values < 0.05 were considered significant.

## Results

### Generation of soluble canine PD-1Ig and PD-L1Ig, and hybridoma development

**Fig 1:**
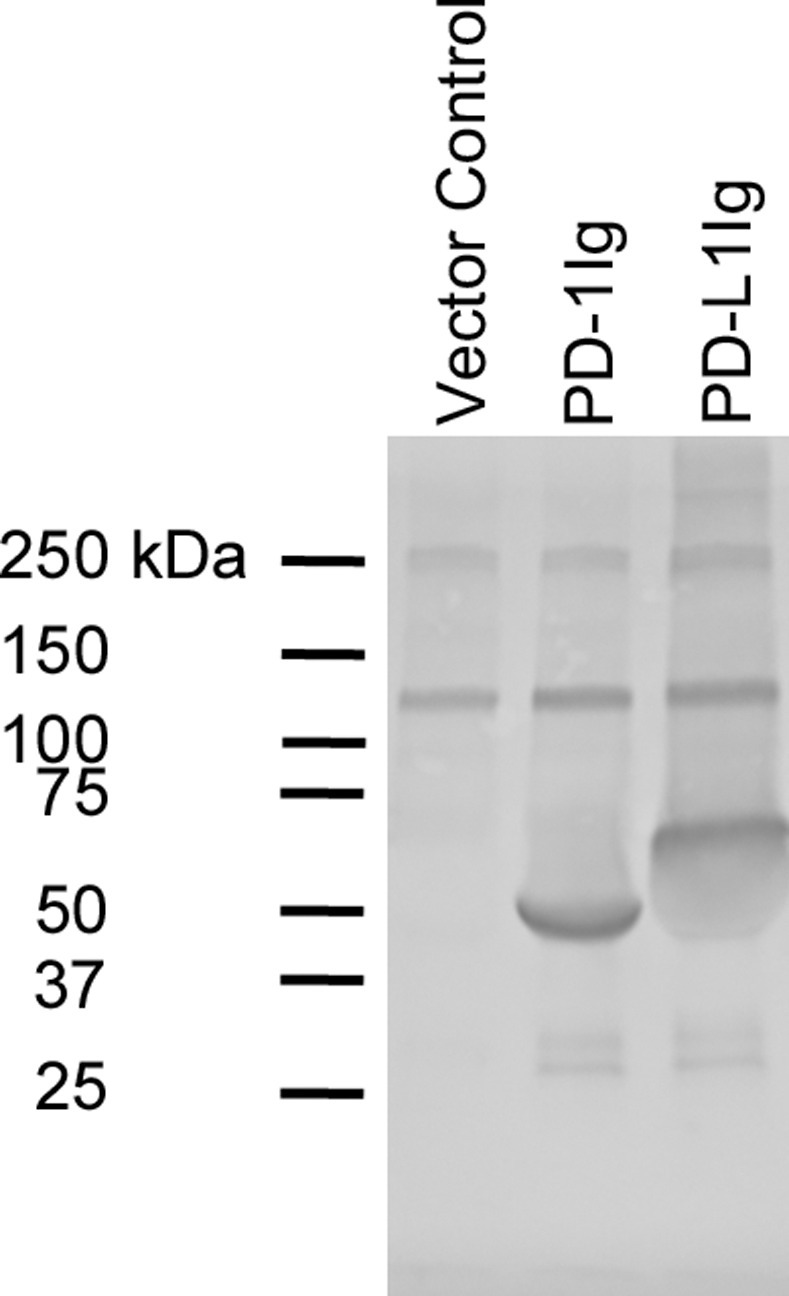
Expression of recombinant PD-1Ig and PD-L1Ig in *D*. *melanogaster* S2 cells. Expression of monomers was tested by Western blot. Biotinylated PD-1Ig and PD-L1Ig were expressed and secreted from *D*. *melanogaster* S2 cells. S2 cell culture supernatant expressing PD-1Ig (lane 2) or PD-L1Ig (lane 3) was analyzed against vector only control (lane 1) on Western blot in reducing condition. Alkaline phosphatase conjugated streptavidin was used to detect proteins.

BALB/c mice were immunized with recombinant PD-1Ig or PD-L1Ig to generate a source of antigen-specific B cells for hybridoma development. To generate canine fusion proteins for immunization, PD-1Ig and PD-L1Ig were co-expressed with BirA, an *E*. *coli* biotin ligase, in S2 cells. PD-1Ig and PD-L1Ig were intracellularly biotinylated by BirA at the biotinylation motif added to the C-terminus of fusion proteins. Correct expression of PD-1Ig and PD-L1Ig was confirmed by Western blot (Fig 1). Each of these fusion proteins was purified from S2 culture supernatants using protein G agarose (Pierce Biotechnology, Rockford, IL). Enriching antigen-specific B-cells from immunized mice was shown to be a successful strategy to increase the frequency of antigen-specific hybridomas and reduce library screening [31]. To take advantage of this approach, canine PD-1Ig and PD-L1Ig tetramers were generated by incubating biotinylated PD-1Ig and PD-L1Ig with PE-conjugated streptavidin (SA-PE). To assure that the majority of biotinylated PD-1Ig or PD-L1Ig was bound to the streptavidin, different ratios of either Ig fusion protein and SA-PE were prepared and compared by Western blot. SA-AP was used to detect unbound biotinylated PD-L1Ig and efficient mixing ratio was confirmed with the absence or very light appearance of PD-1Ig (S1 Fig) or PD-L1Ig band (S2 Fig).

Two immunization approaches were used to generate PD-1 or PD-L1 specific B cells. Sequential subcutaneous injection of exponentially increasing antigen dose (Exp-Inc) in combination with MPLA enhances antibody responses to immunization [30]. Therefore, we immunized an initial cohort of BALB/c mice using this method and a second cohort were immunized with a more traditional single bolus approach using antigen emulsified in CFA. PD-1-, and PD-L1-specific B-cells were enriched by tetramer binding and subsequent MACS purification before fusion with SP2/0 myeloma cells (S3 and S4 Figs). Resulting hybridomas supernatants were tested for PD-1Ig and PD-L1Ig specificity on an ELISA using plate-bound PD-1Ig or PD-L1Ig. Immunization with PD-L1Ig/MPLA (Exp-Inc) generated 127 hybridomas of which 8 were ELISA positive for binding to PD-L1. Immunization with PD-L1Ig/CFA generated 722 hybridomas and 35 were specific for PD-L1. PD-1Ig immunization (Exp-Inc) generated 64 hybridomas but only 1 was positive by PD-1 ELISA. In contrast, PD-1Ig/CFA generated 288 hybridomas with 10 being highly specific for PD-1. The most striking difference between Exp-Inc method and CFA using bolus prime/boost method was that CFA using bolus prime/boost method generated 72 out of 288 PD-1Ig hybridomas and 286 out of 722 PD-L1Ig hybridomas were specific for human IgG1 Fc while Exp-Inc method generated no hybridomas specific for human IgG1 Fc.

### Evaluation of anti-PD-1 and anti-PD-L1 blocking capacity by ELISA

**Fig 2:**
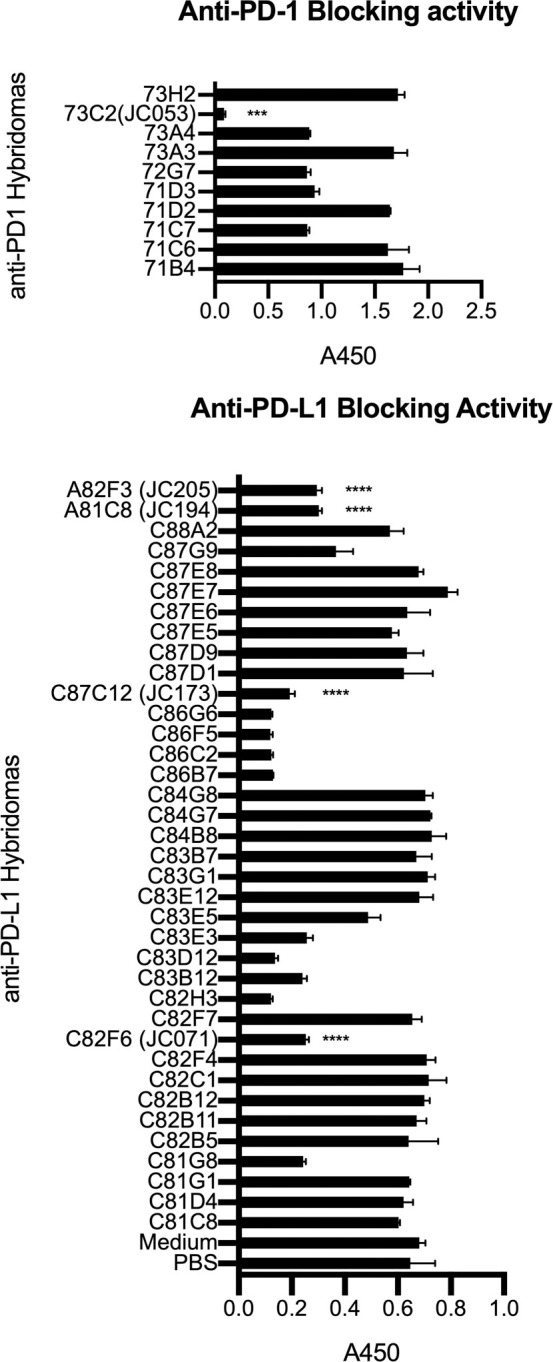
Evaluation of blocking activity by anti-canine PD-1 and PD-L1 antibodies. Antibodies were screened for their opposite ligand blocking capability. Hybridoma supernatants were added to ELISA plates coated with unbiotinylated PD-1Ig (A) or PD-L1Ig (B). After washing, biotinylated PD-L1Ig (A) or PD-1Ig (B) was used to detect blocking capacity of anti-PD-1 (A) and anti-PD-L1 (B) using HRP conjugated streptavidin to detect unblocked protein. All samples were assayed in duplicates. Each value is presented as mean ± standard deviation. In (A), JC053 value was significantly different from the rest of anti-PD-1 hybridomas with P value of 0.0002. In (B), JC071, JC173, JC194, and JC205 values were significantly different from PBS and medium values with P values less than 0.0001.

Next, we developed a blocking ELISA to test whether PD-1 or PD-L1-specific antibodies were able to block binding of PD-1 to PD-L1. Unbiotinylated PD-1Ig-NB and PD-L1Ig-NB were coated on separate ELISA plates. After the application of hybridoma culture supernatant, we assessed whether biotinylated PD-1Ig or PD-L1Ig could bind to the respective plates. The PD-1 specific hybridoma from Exp-Inc immunization did not block PD-1 and PD-L1 interactions, but 5/10 PD-1 specific hybridomas from CFA immunization displayed a range of blocking capability (Fig 2). Only 2/8 PD-L1Ig-specific hybridomas from Exp-Inc immunization blocked binding between PD-L1Ig-NB and PD-1Ig. In contrast, 13/35 hybridomas from CFA immunization produced blocking antibodies (Fig 2). Given the goal of generating functional reagents, only antibodies that were capable of blocking PD-1/PD-L1 interactions were further assessed.

### Antibody binding to recombinant and native PD-1/PD-L1

**Fig 3:**
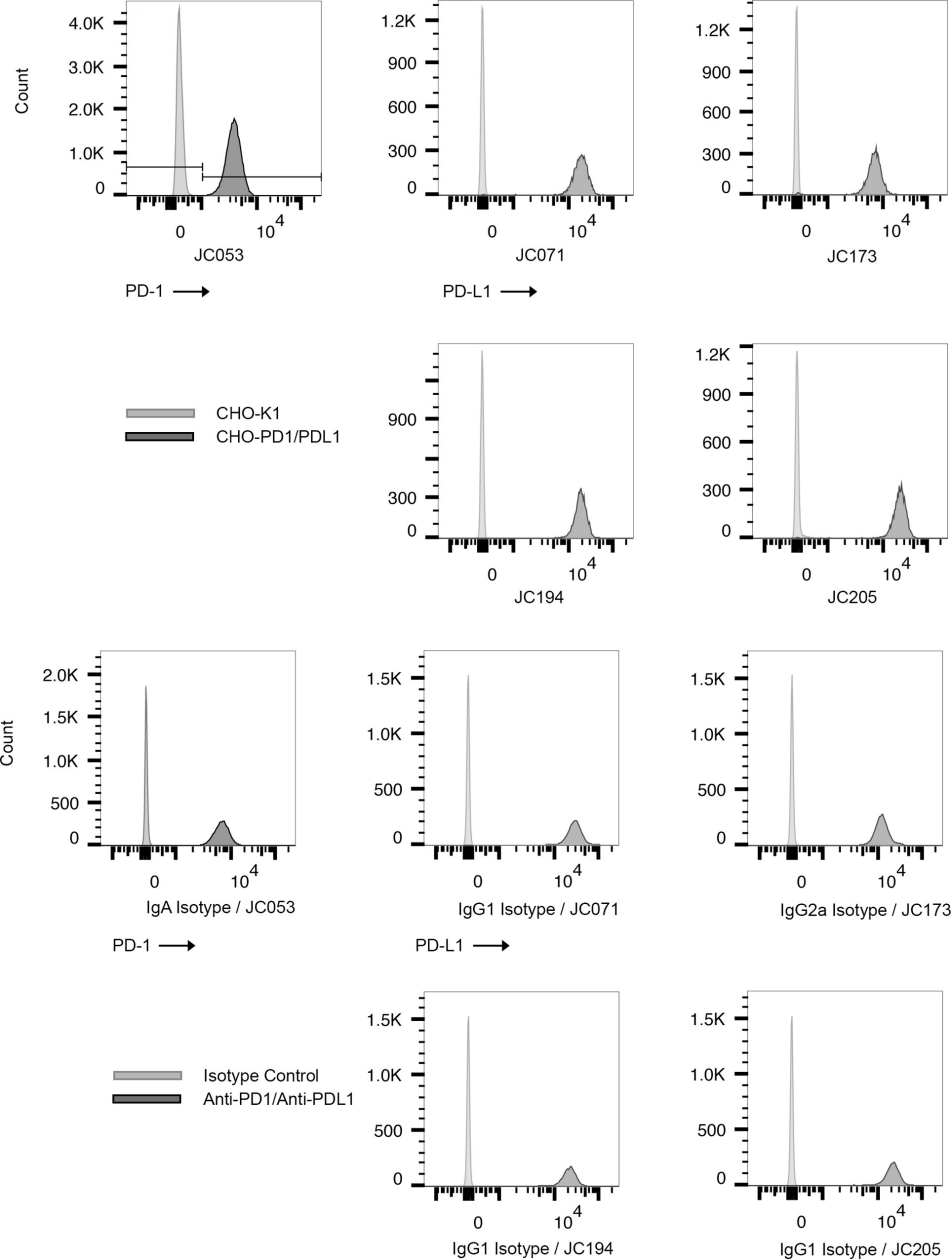
Detection of PD-1 and PD-L1 expression on CHO cells expressing canine markers. Antibody specificity toward PD-1 and PD-L1 by anti-PD-1 (JC053) and anti-PD-L1s (JC071, JC173, JC194, and JC205) was tested on CHO cells by flow cytometry. (A) Anti-PD-1 and anti-PD-L1 bind to CHO-PD1 and CHO-PDL1, respectively, while they do not bind to untransfected CHO-K1. PD-1 positive population were selected following FSC, SSC gating, single cell gating, and live cell gating. PD-L1 positive population was selected following FSC, SSC gating, and single cell gating. (B) JC053, an anti-PD-1 antibody, was compared to a mouse IgA isotype control for staining CHO-PD1. Biotinylated anti-mouse IgA and APC conjugated streptavidin was used following primary antibody staining. A population of CHO-PD1 cells was selected on FSC and SSC gating. Single cells were selected and dead cells were excluded. Then, PD-1 positive cells are shown on histograms. Anti-PD-L1s were conjugated with PE and used to stain CHO-PDL1 cells. Appropriate isotype controls were included and anti-mouse IgG1 and anti-mouse IgG2a were used in combination with PE conjugated streptavidin. PE positive population is shown on histograms following FSC/SSC gating, single cell selection, and exclusion of dead cells.

**Fig 4:**
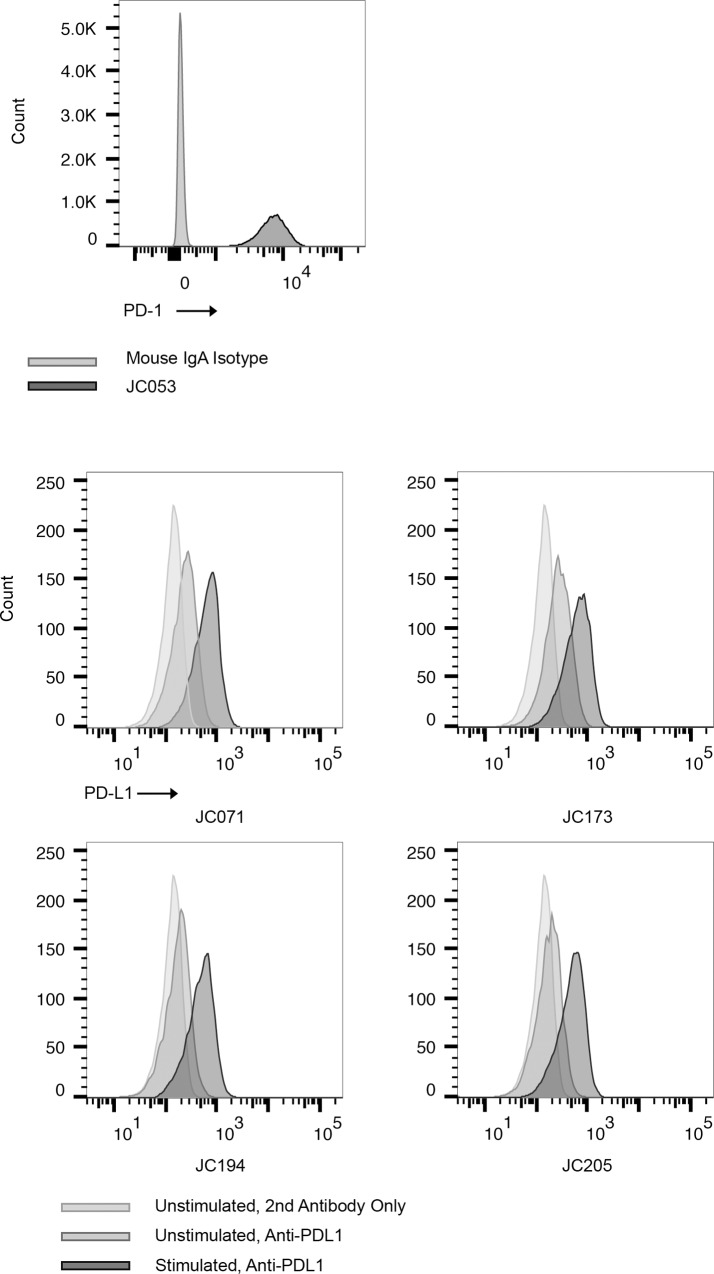
Detection of PD-1 and PD-L1 expression on canine cell lines. (A) PD-1 expression on CLGL-90 cells was detected with JC053 hybridoma culture supernatant. Biotinylated anti-mouse IgA and FITC-conjugated streptavidin was used. Mouse IgA isotype control was used as negative control. Light gray is for isotype control and dark gray is for JC053. (B) Four purified anti-PD-L1s were compared in their capacity to bind activated DH82 cells. DH82 cells were cultivated either with or without 10ng/ml IFN-γ and JC071, JC173, JC194, and JC205 binding to PD-L1 was assessed. Histograms show unstimulated DH82 without anti-PD-L1 (light gray), unstimulated DH82 with anti-PD-L1 (medium gray), and IFN-γ stimulated DH82 with anti-PD-L1 (dark gray), respectively. FITC conjugated Anti-Mouse IgG was used with all samples including no anti-PD-L1 sample, as secondary antibody. PD-1 or PD-L1 positive population was selected following FSC, SSC gating, single cell gating, and live cell gating.

To determine whether these antibodies could bind PD-1 or PD-L1 expressed on cell surfaces, CHO-K1 cells were transfected with either PD-1 or PD-L1 to generate CHO-PD1 and CHO-PDL1, respectively. We used CHO-PD1 cells to evaluate PD-1 antibodies, and CHO-PDL1 cells to evaluate PD-L1 antibodies by flow cytometry. Based on initial screening with culture supernatants, we selected a single PD-1 specific hybridoma and 4 PD-L1 specific hybridomas for sub-cloning by limiting dilution and an initial analysis of antibody isotype. Results of these tests supported further consideration for the following monoclonal antibodies; JC053 anti-PD-1 (IgA, CFA), JC071 anti-PD-L1 (IgG1, CFA), JC173 anti-PD-L1 (IgG2a, CFA), JC194 anti-PD-L1 (IgG1, Exp-Inc), and JC205 anti-PD-L1 (IgG1, Exp-Inc). JC053 was able to detect canine PD-1 expression on CHO cells, while the other four antibodies detected canine PD-L1 expression on CHO cells by flow cytometry (Fig 3). Antibody binding to PD-1 or PD-L1 on CHO cells is specific to PD-1 or PD-L1 since these antibodies did not bind to untransfected CHO-K1 (Fig 3A). Next, we assessed whether these antibodies would bind native antigen expressed by canine cell lines. CLGL-90 is a canine large granular T-cell leukemia cell line that expresses PD-1 [35]. Clone JC053 proved capable of detection for cell surface expression of PD-1 on CLGL-90 cells by flow cytometry (Fig 4A). DH82 is a histiocytic sarcoma cell line that normally expresses PD-L1 at basal level but enhanced level of PD-L1 following stimulation [28]. All four monoclonal antibodies specific for PD-L1 were able to detect expression of PD-L1 on DH82 both with and without *in vitro* activation using IFN-γ (Fig 4B). Thus, all five selected monoclonal antibodies efficiently bound recombinantly expressed PD-1 or PD-L1 on CHO cells, and to natively expressed PD-1 or PD-L1 on canine cell lines.

### Detection of PD-1-, and PD-L1-expressing cells in canine peripheral blood

**Fig 5:**
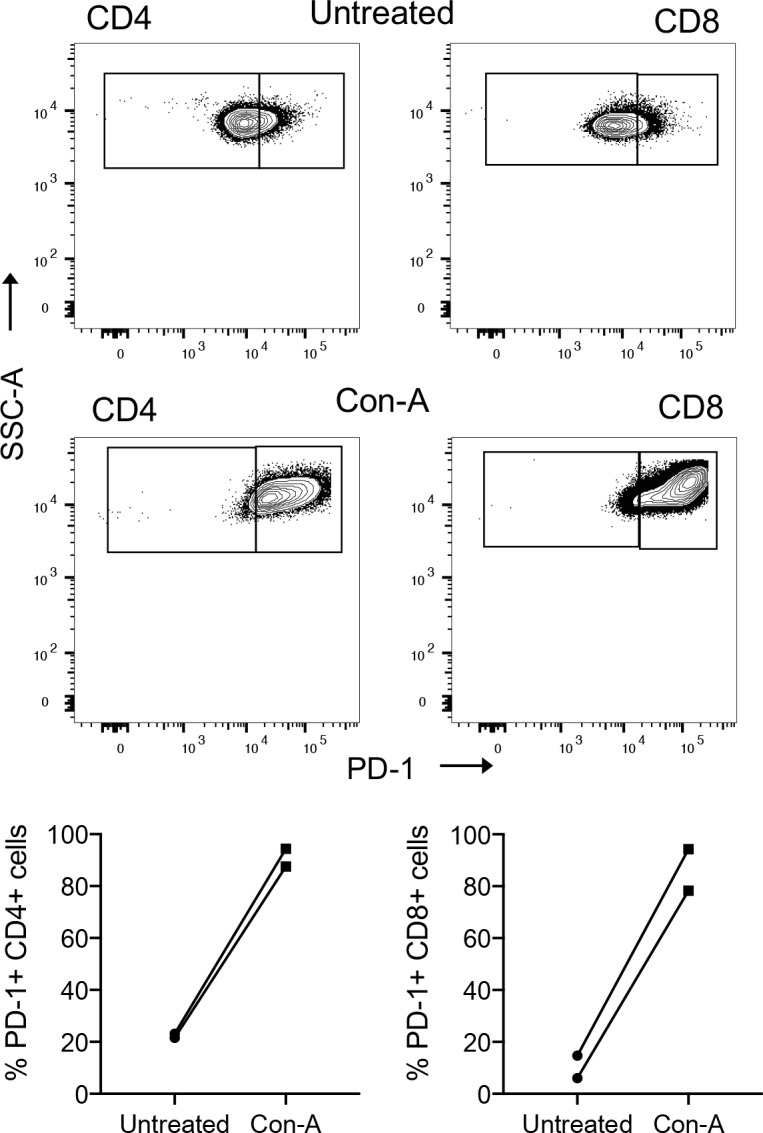
Detection of PD-1 expression on CD4^+^ and CD8^+^ T-cells from peripheral blood by anti-canine PD-1 antibody JC053. Representative staining of PBMC T cell subsets for PD-1 expression by flow cytometry using antibody JC053 from a healthy canine donor is shown before and after stimulation with ConA for 24 hours (A). Frequencies of PD-1 expressing CD4^+^ and CD8^+^ T-cells before and after stimulation of PBMCs from two healthy canine donors were evaluated by flow cytometry using antibody JC053.

The data above demonstrated that the antibodies efficiently detected PD-1 or PD-L1 on recombinant cells and selected canine cell lines. Therefore, we next examined these reagents for characterization of PD-1 or PD-L1 expression on primary canine PBMC subsets. First, unstimulated and ConA-stimulated PBMCs were examined for PD-1 expression by flow cytometry analysis. PBMCs were first interrogated with anti-human CD3 antibody followed by anti-canine CD4 and CD8 antibodies using a gating strategy (S5 Fig) as previously described [32] and subsequently analyzed for PD-1 expression using the JC053 anti-canine PD-1 antibody. Low frequencies of PD-1^+^ cells were detected for both CD4^+^ and CD8^+^ T cell subsets in the absence of stimulation with a positive frequency range of 6 to 23% (Fig 5). As expected, stimulation with ConA generated increased frequencies of PD-1^+^ CD4^+^ and CD8^+^ T cells (78–94%) (Fig 5).

**Fig 6:**
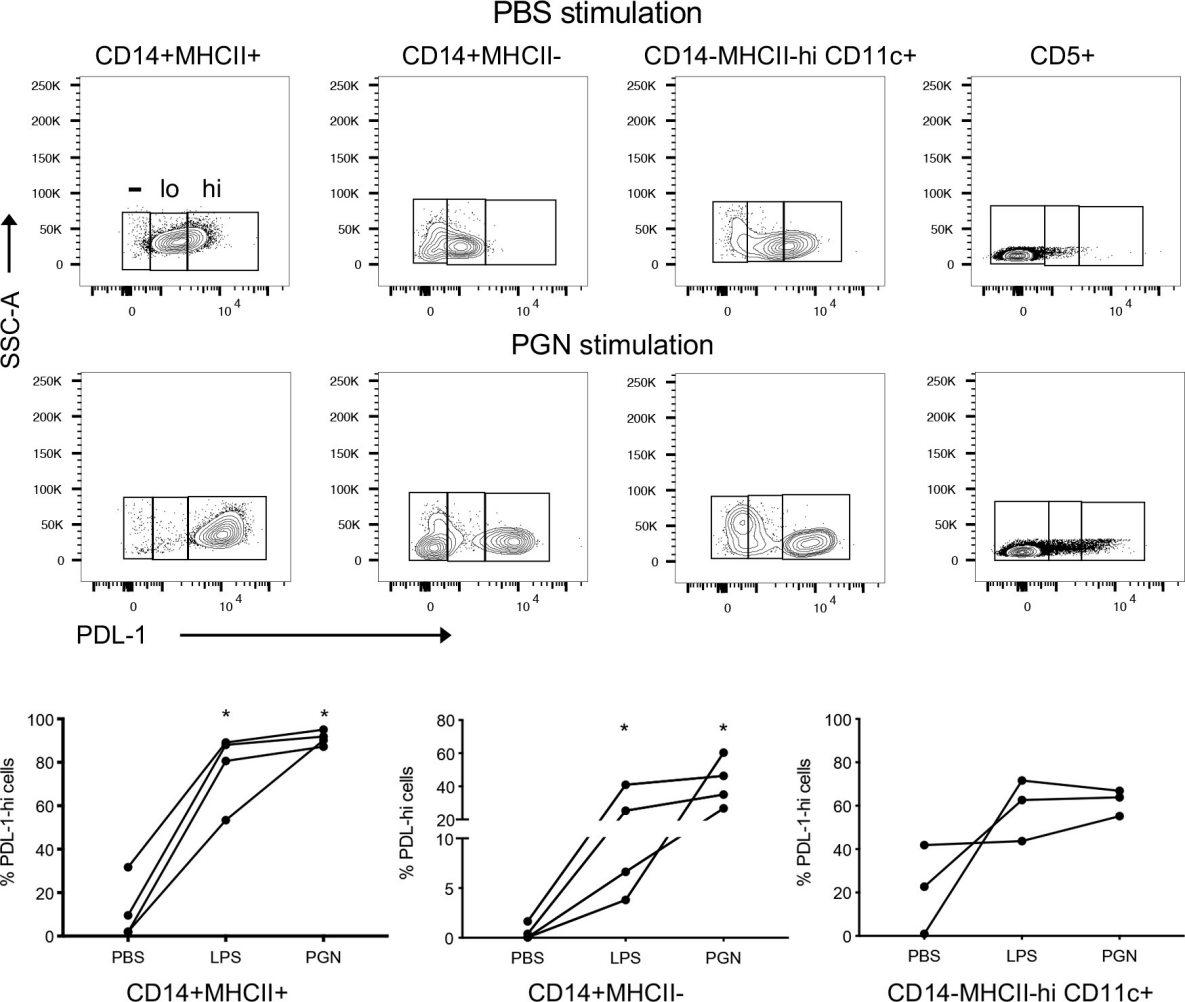
Detection of PD-L1 expression on peripheral blood immune cells by anti-canine PD-L1 antibody JC071. PBMCs from healthy donor dogs were stimulated with PBS, LPS, or PGN for 3 hours followed by overnight incubation. A representative staining of PBMC immune cell subsets for PD-L1 expression by flow cytometry using antibody JC071 from a healthy canine donor is shown before and after stimulation with PGN (A). Frequencies of monocyte subsets (MHCII^+^ CD14^+^ and MHCII^-^CD14^+^) from four canine donors and MHCII^hi^ CD14^-^ CD11c^+^ dendritic cells from three donors (B). A pair-wise comparison between stimulation conditions by the Mann-Whitney test was used for statistical analysis. Statistically significant differences are indicated by an astericks.

Using a gating strategy shown in S6 Fig, monocytes were defined as CD5^-^CD14^+^ PBMC and further characterized by canine MHCII expression (MHCII^+^ or MHCII^-^). Dendritic cells (DC) were defined as CD5^-^CD14^-^ PBMC demonstrating high expression of MHCII (MHCII^hi^) and expression of CD11c (CD11c^+^) (S6 Fig). Monocyte subsets and DC were characterized for PD-L1 expression and stratified into PD-L1^-^, PD-L1^low^, PD-L1^hi^ populations using the JC071 anti-canine PD-L1 antibody as shown by representative PBMC staining in Fig 6A and based on analysis with an isotype control (S7 Fig). Frequencies of PD-L1^hi^ populations within healthy canine donor PBMCs treated with either PGN, LPS, or PBS, were analyzed for monocyte subsets (MHCII^+^ and MCHII^-^) and DC (Fig 6B). Unstimulated MHCII^+^CD14^+^ monocytes revealed a variable frequency of PD-L1^+^ cells (2%-34%); frequencies were markedly increased (78–95%) upon LPS or PGN stimulation (Fig 6B). Unstimulated MHCII^-^CD14^+^ monocytes exhibited lower and more variable frequencies (0.04–2.0%) of PD-L1^+^ cells. However, stimulation resulted in 16–1,700-fold increases in PD-L1^+^ cell frequencies (Fig 6A). DC also displayed a trend towards increased PD-L1^+^ cell frequencies after LPS and PGN stimulation, although this increase was not statistically significant and more variable (Fig 6B). Overall, both LPS and PGN led to a marked increase in the frequency of PD-L1^+^ cells in canine PBMC monocyte populations, with PGN frequently showing stronger induction of PD-L1 expression compared to LPS. Importantly, expected staining patterns for both PD-1 and PD-L1 expression were observed in PBMC subsets after conditions of stimulation with the use of anti-canine PD-1 (JC053) and anti-canine PD-L1 (JC071) monoclonal antibodies.

### PD-1 antibody staining of canine tissues

**Fig 7:**
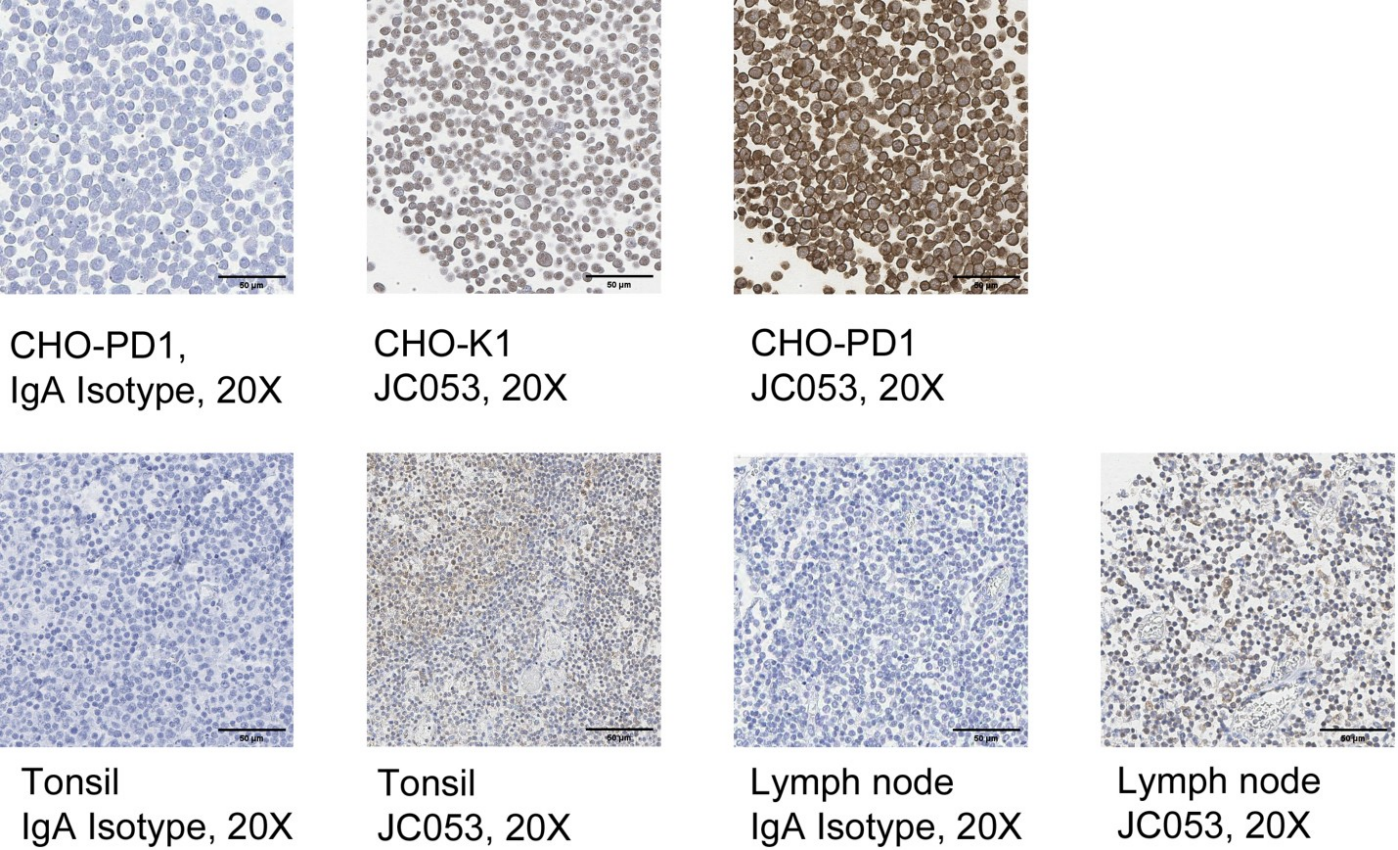
Detection of PD-1 expression on formalin-fixed cells and FFPE tissues by anti-canine antibody JC053. JC053 was tested for evaluation of PD-1 expression on formalin-fixed cells as well as canine tissues. CHO-K1 as well as CHO-PD1 cells (A) were fixed with formalin, treated with Histogel and paraffin blocks were prepared. Cells were stained with JC053 (1:100 dilution) and compared to IgA isotype control stained cells (1:100 dilution) in IHC. FFPE tonsil and lymph node (B) were also evaluated for PD-1 expressions. JC053 was diluted at 1:1000 and IgA isotype control was diluted at 1:100. Images for JC053 are 40x magnified and IgA isotype control images are 20x magnified.

Since JC053 was able to detect PD-1 on CHO-PD1 cells, as well as canine PBMC, we tested the utility of JC053 to detect PD-1 on histological sections. Initially, CHO-PD1 and untransfected CHO-K1 cells were prepared in Histogel cell blocks and formalin fixed before staining with JC053. A murine IgA isotype control failed to show any background staining on these blocks or subsequent canine tissues (Fig 7A). Although JC053 displayed some faint background staining on CHO-K1 cells, a much stronger signal was evident on CHO-PD1 cells (Fig 7A), suggesting that the antibody was detecting PD-1 in these tissue sections. JC053 concentration for CHO-K1 and CHO-PD1 cell block staining was 10-fold higher than for lymph node and tonsile tissue staining. This high concentration of JC053 could have led to high background staining on CHO-K1. Encouraged by this result, we examined whether JC053 could detect PD-1 in formalin-fixed canine tissues. Indeed, JC053 successfully stained tonsil and lymph node sections (Fig 7B), indicating that this antibody can be used for histological detection of canine PD-1.

### Amplification of IFN-γ production as a consequence of PD-L1 blockade

**Fig 8:**
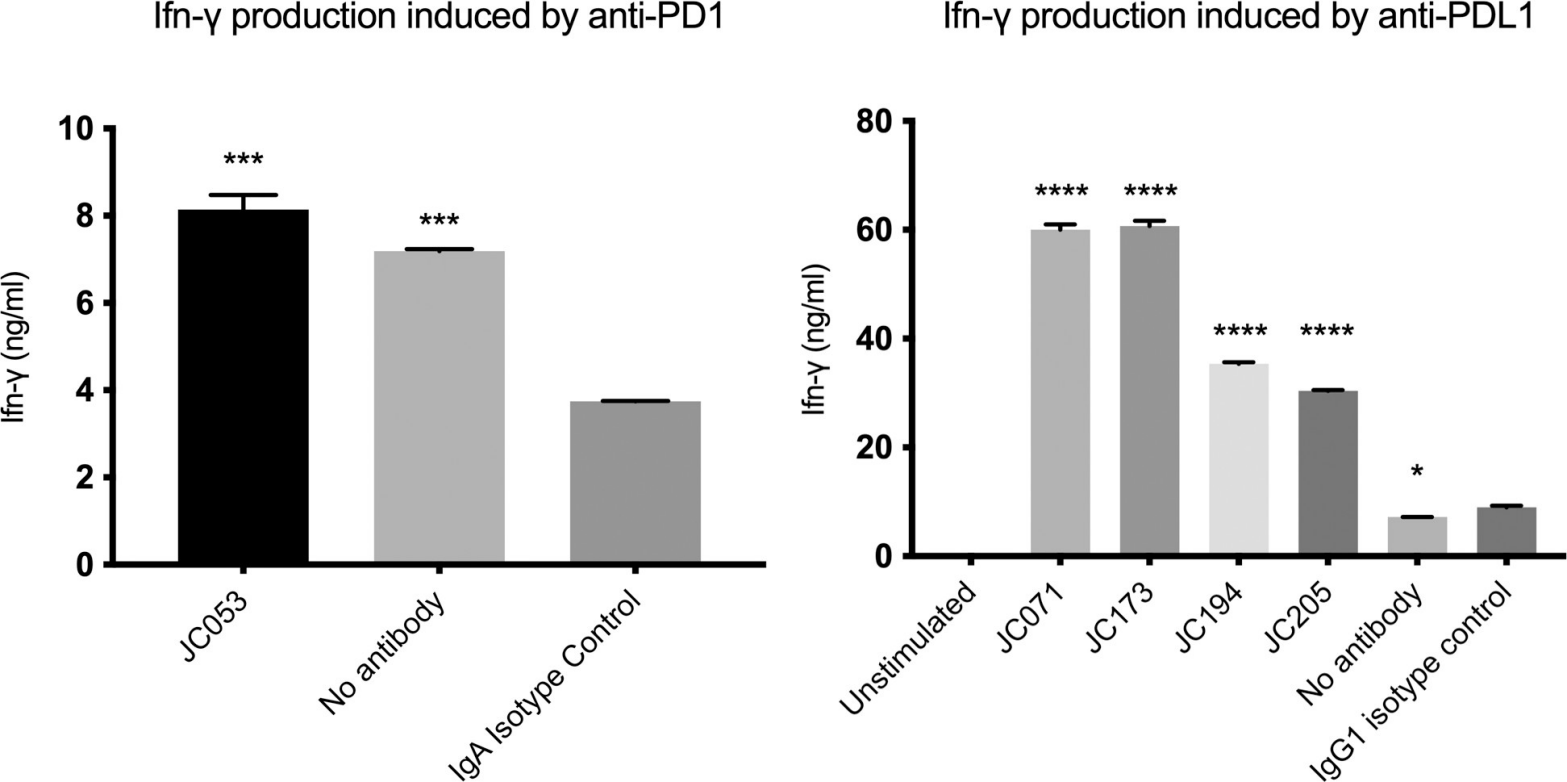
Amplification of IFN-γ production from PBMC via PD-1 / PD-L1 blockade. PD-1 and PD-L1 blocking by antibodies led to increased IFN-γ production in PBMC culture compared to isotype controls. PBMCs from a healthy canine donor were stimulated with 1 ug/ml ConA in the presence or absence of 0.2 uM monoclonal antibodies. IFN-γ produced by stimulated cells were assessed on ELISA. (A) JC053 blocked cells produced significantly higher IFN-γ compared to IgA isotype control but was not significantly different from no antibody control. (B) All four anti-PD-L1 samples produced significantly higher IFN-γ compared to IgG1 isotype control. Results are from duplicates. P-value was either < 0.0001 (****), <0.001 (***), or < 0.05 (*).

Introduction of PD-1/PD-L1 blockade can recover functional activity of T cells in *in vitro* and *in vivo* assays [36]. Maekawa et al. has shown that addition of anti-PD-L1 in dog PBMC culture increases Ifn-γ level [29]. Although similar experiment by Nemoto et al. did not lead to significantly increased Ifn-γ, it showed the increasing trend [27]. Thus, to determine whether blockade of PD-L1 and PD-1 interactions by any of these five monoclonal antibodies could lead to enhanced T-cell activity, we generated a low dose ConA stimulation assay for canine PBMC cultures and examined the capacity to enhance IFN-γ production. Although the addition of JC053 led to more than 2-fold higher IFN-γ production compared to an IgA isotype control, this was not significantly different from no antibody controls (Fig 8A). Thus, JC053 does not cause a recovery of T cell function in this assay. In marked contrast, JC194 and JC205 enhanced IFN-γ production by more than 3-fold compared to an IgG1 isotype control, as well as no antibody control after 96 hours stimulation of canine PBMCs (Fig 8B). Similarly, JC071 and JC173 amplified IFN-γ production in culture by approximately 6-fold (Fig 8B). This indicates that all 4 PD-L1 blocking antibodies alleviated T-cell suppression and led to enhanced functional activity of T cells in culture.

## Discussion

Standard treatment methods for canine cancer involve surgical, radiation, and chemotherapeutic approaches, much like human medicine. Human cancer therapy approaches are rapidly changing due to the successes of immunotherapy using drugs such as ipilimumab (anti-CTLA4), pembrolizumab (anti-PD-1), atezolizumab (anti-PD-L1), and tisagenlecleucel (CD19 CAR-T). These successful interventions should guide canine cancer strategies to develop more effective therapies for use in veterinary medicine. The development of canine immune therapeutic approaches could also expand comparative oncology approaches to improve our understanding of human disease and the future therapeutic potential of these agents. Unique factors such as breed genetic similarity, spontaneous canine tumor development in the face of an intact immune system, the similar microbiota of cohabiting dogs and humans, and the correlation between advanced age and tumor onset, strongly suggest that canine immunotherapy could inform human medicine especially as combination therapies and dosing schedules are refined [2]. In fact, several canine-specific reagents have been developed and have shown to be effective *in vitro*, as well as in a few limited canine clinical studies. Importantly, these canine reagents include anti-PD-1, anti-PD-L1, CD20 CAR-T therapy, and NK-cell therapy, all approaches that have shown success in human medicine [7,25,27,37–39]. As yet, none of these reagents are commercially available for use in canine patients and thus additional reagent development is warranted.

We have developed new canine PD-1 and PD-L1-specific monoclonal antibodies for development as potential therapeutic agents, as well as diagnostic purposes. Our PD-1/PD-L1 antibodies were generated using a mouse hybridoma methodology that has been adapted to include B cell enrichment to facilitate rapid selection of specific clones. Traditional hybridoma approaches usually require screening of hundreds to thousands of candidates clones in order to find appropriate antigen specific clones. Incorporating a protein-tetramer B-cell enrichment strategy prior to fusion has been previously shown to be useful for reducing the number of unwanted irrelevant clones [31]. Our JC053 and JC071 clones were obtained after 50-fold and 5-fold enrichment of specific B cells by tetramer-assisted MACS purification (S3 and S4 Figs). However, naïve B cells were also enriched by around 2-fold and 4.4-fold respectively using the PD-1 tetramer and PD-L1 tetramers. After considering this enrichment effect of naïve samples, the use of a PD-1 tetramer resulted in a 25-fold normalized enrichment effect, while the PD-L1 enrichment was much lower, around 1.14-fold. It is not yet clear why there was 20-fold difference in these enrichment approaches or why our PD-L1 tetramer bound strongly to naïve cells. However, these data suggest that including tetramer enrichment can enhance the selection of positive B cell clones in at least some cases.

In this study, we also used two different immunization methods to develop monoclonal antibodies specific for canine PD-1 and PD-L1. The first approach used seven consecutive injections of antigen in an exponentially increasing dose in combination with MPLA as an adjuvant. This method is intended to mimic a natural infection where infecting microorganisms grow exponentially inside the host and stimulate a more natural and robust immune response. The second approach was a traditional prime boost strategy using CFA as an adjuvant. Considering that both recombinant antigens were fused to a human IgG1 Fc region, the expectation would be that human Fc-specific hybridomas would be detected as a result of both immunizations. To our surprise, our immunization using the exponentially increasing dosing strategy generated no human Ig-specific hybridomas, while the CFA single bolus injection approach resulted in the majority of hybridomas being Fc specific. While this outcome would need to be tested in a more systematic manner, it would appear that the use of an MPLA/exponentially increasing dosing method may have led to the generation of more antigen specific hybridomas.

PD-1 and PD-L1 antibodies have been shown to have diagnostic predictive value in certain human cancers [40,41]. As a first step towards a diagnostic approach in dogs, we examined the utility of these antibodies by a variety of immunological approaches. All five antibodies described herein successfully detected recombinant purified PD-1 or PD-L1 or the same molecules expressed on transfected CHO-K1 cells by ELISA. These antibodies were also capable of binding canine PD-1 and PD-L1 expressed on both canine cell lines and primary canine immune cell subsets under conditions of stimulation in a predictable manner using flow cytometry analysis. Additionally, two of these antibodies (JC053 and JC071) successfully detected PD-1 (S8 Fig) and PD-L1 (S9 Fig) by Western blot. Finally, JC053 successfully detected canine PD-1 expressed on formalin-fixed paraffin-embedded (FFPE) tonsil and lymph node suggesting that this could be useful as diagnostic tool in tumor biopsies. Since the clinical response rate of PD-1/PD-L1 therapy in humans varies from slightly above 10% to over 80% in different tumors and clinical studies, there is considerable interest in using the patient tumor microenvironment to predict treatment outcome. In certain human tumors, high expression of PD-L1 on tumor cells is associated with a good prognosis [42], while the presence of M2 macrophages in the tumor environment is associated with tumor resistance [43–45]. In other cases, PD-L1 expression on host immune cells is associated with a favorable prognosis [42]. Since these reagents are not yet available to routinely assess canine tumor biopsies, development of these reagents (JC053 and JC071) could contribute to more accurate prognosis of progression or treatment response rates for certain canine cancers. Additional studies to examine this possibility are under consideration.

Most importantly, our data demonstrate that all four anti-canine PD-L1 antibodies significantly increased IFN-γ production in a PBMC assay, where T cells were stimulated by low dose Con A compared to isotype control samples. These results show considerable promise since they suggest that these antibodies are capable of reviving T-cell responses in a context where PD-1/PD-L1 inhibition occurs. However, clinical use of these antibodies as a viable therapeutic reagent will require further engineering to remove the murine framework sequences to avoid a strong anti-drug response leading to premature clearance of the antibodies. To avoid this problem, the framework of current antibodies can be changed to match a canine antibody framework using a chimeric antibody approach or by incorporating the canine Fv framework sequences in addition to the constant domains. However, our functional data suggest that such an approach could yield considerable clinical benefit *in vivo*. Strategies to engineer an appropriate therapeutic intervention based on these antibody specificities are currently underway.

## Conclusion

In this report, we described our efforts to develop monoclonal antibodies against canine PD-1 and PD-L1. All of our antibodies worked effectively on ELISA and flow cytometry applications while JC053 and JC071 also worked on Western blot. Additionally JC053 effectively detected PD-1 by immunohistochemistry of formalin-fixed paraffin embedded tissues. Importantly, JC071 and JC173 showed functional activity in a canine PBMC Ifn-γ assay and therefore have potential application as an immunotherapy reagent for cancer treatment. Current cancer treatment dogs have limited immunotherapy options. Development of more immunotherapy reagents such as our canine specific PD-1 and PD-L1 antibodies will benefit progress of cancer research as well as the treatment of canine patients.

## Supporting information

S1 Table List of client owned dogs used in this study. (DOCX)

S2 Table List of antibodies used in current study. (DOCX)

S1 Fig PD-1Ig tetramer generation. Each monomer was mixed with SA-PE to generate tetramer. To find correct ratio between PD-1Ig and SA-PE for tetramer generation, different ratios of monomer and SA-PE were tested on Western blot in non-reducing condition. Samples are monomer only (lane 1), SA-PE only (lane 2), monomer and SA-PE with 1:1 (lane 3), 2.5:1 (lane 4), 5:1 (lane 5), 10:1 (lane 6), and 20:1 molar ratio (lane 7). Correct ratio was determined to be 2.5:1 ratio for both PD-1Ig.
(TIF)

S2 Fig PD-L1 tetramer generation. Each monomer was mixed with SA-PE to generate tetramer. To find correct ratio between PD-L1Ig and SA-PE for tetramer generation, different ratios of monomer and SA-PE were tested on Western blot in non-reducing condition. Samples are monomer only (lane 1), SA-PE only (lane 2), monomer and SA-PE with 1:1 (lane 3), 2.5:1 (lane 4), 5:1 (lane 5), 10:1 (lane 6), and 20:1 molar ratio (lane 7). Correct ratio was determined to be 2.5:1 ratio for both PD-L1Ig.
(TIF)

S3 Fig PD-1Ig tetramer-aided B-cell enrichment efficiency. Dump- Tetramer+ frequency for PD-1Ig immunized sample can be compared to naïve sample when PD-1Ig tetramer was applied.
(TIF)

S4 Fig PD-L1Ig tetramer-aided B-cell enrichment efficiency. Dump Tetramer^+^ frequency for PD-L1Ig immunized sample can be compared to naïve sample when PD-L1Ig tetramer was applied.
(TIF)

S5 Fig Gating strategy for CD4^+^ and CD8^+^ T cells. A standard gating strategy used for CD4^+^ and CD8^+^ T cell subsets by flow cytometry and for analysis of frequencies of PD-1^+^ populations is shown.
(TIF)

S6 Fig Gating strategy for monocytes and dendritic cells after staining with JC071. The basic gating strategy used for immune cell subsets by flow cytometry and for analysis of frequencies of PD-L1^+^ populations is shown. Subsets of interest included CD5^-^MHCII^+^CD14^+^ and CD5^-^MHCII^-^CD14^+^ monocytes and DC defined as CD5^-^MHCII^hi^CD14^-^CD11c^+^.
(TIF)

S7 Fig CD5-MHCII+CD14+ monocyte subset isotype control staining. Staining of the CD5^-^MHCII^+^CD14^+^ subset before and after PGN stimulation with an isotype control antibody is also shown.
(TIF)

S8 Fig Application of JC053 in Western blot. Soluble PD-1Ig was detected on Western blot in non-reducing condition using JC053, and anti-mouse IgG-AP, sequentially (Right). This was compared to biotinylated PD-1Ig detected using SA-AP (Left). Two blots using SA-AP and JC053 were prepared on separate membranes.
(TIF)

S9 Fig Application of JC071 in Western blot. Soluble PD-L1Ig expressed in S2 was detected on Western blot in non-reducing condition using JC071 and anti-mouse IgG-AP, sequentially (Right). This was again compared to SA-AP treated blot (Left). Two blots using SA-AP and JC071 were prepared on separate membranes.
(TIF)

S1 Raw images (PDF)

## References

[1] Schiffman JDD, Breen M. Comparative oncology: what dogs and other species can teach us about humans with cancer. Phil Trans R Soc B. 2015;370(1673):20140231 10.1098/rstb.2014.0231 26056372 PMC4581033

[2] Park JS, Withers SS, Modiano JF, Kent MS, Chen M, Luna JI, et al Canine cancer immunotherapy studies: linking mouse and human. J Immunother Cancer. 2016 12 20;4(1):97 10.1186/s40425-016-0200-7 28031824 PMC5171656

[3] Maude SL, Laetsch TW, Buechner J, Rives S, Boyer M, Bittencourt H, et al Tisagenlecleucel in Children and Young Adults with B-Cell Lymphoblastic Leukemia. N Engl J Med. 2018;378(5):439–48. 10.1056/NEJMoa1709866 29385370 PMC5996391

[4] Schadendorf D, Hodi FS, Robert C, Weber JS, Margolin K, Hamid O, et al Pooled analysis of long-term survival data from phase II and phase III trials of ipilimumab in unresectable or metastatic melanoma. J Clin Oncol. 2015;33(17):1889–94. 10.1200/JCO.2014.56.2736 25667295 PMC5089162

[5] Hamid O, Robert C, Daud A, Hodi FS, Hwu WJ, Kefford R, et al Safety and tumor responses with lambrolizumab (anti-PD-1) in melanoma. N Engl J Med. 2013;369(2):134–44. 10.1056/NEJMoa1305133 23724846 PMC4126516

[6] Schmid P, Adams S, Rugo HS, Schneeweiss A, Barrios CH, Iwata H, et al Atezolizumab and nab-paclitaxel in advanced triple-negative breast cancer. N Engl J Med. 2018 11;379(22):2108–21. 10.1056/NEJMoa1809615 30345906

[7] Addissie S, Klingemann H. Cellular immunotherapy of canine cancer. Vet Sci. 2018;5(4):100. 10.3390/vetsci5040100 PMC6313932 30563208

[8] June CH, Sadelain M. Chimeric antigen receptor therapy. N Engl J Med. 2018;379(1):64–73. 10.1056/NEJMra1706169 29972754 PMC7433347

[9] Ribas A, Wolchok JD. Cancer immunotherapy using checkpoint blockade. Science. 2018;359(6382):1350–5. 10.1126/science.aar4060 29567705 PMC7391259

[10] Ying Z, Huang XF, Xiang X, Liu Y, Kang X, Song Y, et al A safe and potent anti-CD19 CAR T cell therapy. Nat Med. 2019 6 22;25(6):947–53. 10.1038/s41591-019-0421-7 31011207 PMC7518381

[11] Wei SC, Duffy CR, Allison JP. Fundamental mechanisms of immune checkpoint blockade therapy. Cancer Discov. 2018;8(9):1069–86. 10.1158/2159-8290.CD-18-0367 30115704

[12] Freeman GJ, Long AJ, Iwai Y, Bourque K, Chernova T, Nishimura H, et al Engagement of the PD-1 immunoinhibitory receptor by a novel B7 family member leads to negative regulation of lymphocyte activation. J Exp Med. 2000;192(7):1027–34. 10.1084/jem.192.7.1027 11015443 PMC2193311

[13] Baumeister SH, Freeman GJ, Dranoff G, Sharpe AH. Coinhibitory Pathways in Immunotherapy for Cancer. Annu Rev Immunol. 2016;34(1):539–73. 26927206 10.1146/annurev-immunol-032414-112049

[14] Gordon SR, Maute RL, Dulken BW, Hutter G, George BM, McCracken MN, et al PD-1 expression by tumour-associated macrophages inhibits phagocytosis and tumour immunity. Nature. 2017 5 17;545(7655):495–9. 10.1038/nature22396 28514441 PMC5931375

[15] Pesce S, Greppi M, Grossi F, Del Zotto G, Moretta L, Sivori S, et al PD/1-PD-Ls checkpoint: Insight on the potential role of NK cells. Front Immunol. 2019 6 4;10(JUN). 10.3389/fimmu.2019.01242 31214193 PMC6557993

[16] Keir ME, Butte MJ, Freeman GJ, Sharpe AH. PD-1 and Its Ligands in Tolerance and Immunity. Annu Rev Immunol. 2008;26(1):677–704. 10.1146/annurev.immunol.26.021607.090331 18173375 PMC10637733

[17] Hui E, Cheung J, Zhu J, Su X, Taylor MJ, Wallweber HA, et al T cell costimulatory receptor CD28 is a primary target for PD-1-mediated inhibition. Science. 2017 3 31;355(6332):1428–33. 10.1126/science.aaf1292 28280247 PMC6286077

[18] Kamphorst AO, Wieland A, Nasti T, Yang S, Zhang R, Barber DL, et al Rescue of exhausted CD8 T cells by PD-1–targeted therapies is CD28-dependent. Science. 2017 3 31;355(6332):1423–7. 10.1126/science.aaf0683 28280249 PMC5595217

[19] Xin Yu J, Hodge JP, Oliva C, Neftelinov ST, Hubbard-Lucey VM, Tang J. Trends in clinical development for PD-1/PD-L1 inhibitors. Nat Rev Drug Discov. 2020 11 4;19(3):163–4. 10.1038/d41573-019-00182-w 32127660

[20] Chen L, Han X. Anti–PD-1/PD-L1 therapy of human cancer: past, present, and future. J Clin Invest. 2015;125(9):3384–91. 10.1172/JCI80011 26325035 PMC4588282

[21] Rittmeyer A, Barlesi F, Waterkamp D, Park K, Ciardiello F, von Pawel J, et al Atezolizumab versus docetaxel in patients with previously treated non-small-cell lung cancer (OAK): a phase 3, open-label, multicentre randomised controlled trial. Lancet. 2017;389(10066):255–65. 10.1016/S0140-6736(16)32517-X 27979383 PMC6886121

[22] Rosenberg JE, Hoffman-Censits J, Powles T, Van Der Heijden MS, Balar A V., Necchi A, et al Atezolizumab in patients with locally advanced and metastatic urothelial carcinoma who have progressed following treatment with platinum-based chemotherapy: A single-arm, multicentre, phase 2 trial. Lancet. 2016;387(10031):1909–20. 10.1016/S0140-6736(16)00561-4 26952546 PMC5480242

[23] Motzer RJ, Penkov K, Haanen J, Rini B, Albiges L, Campbell MT, et al Avelumab plus axitinib versus sunitinib for advanced renal-cell carcinoma. N Engl J Med. 2019;380(12):1103–15. 10.1056/NEJMoa1816047 30779531 PMC6716603

[24] Antonia SJ, Villegas A, Daniel D, Vicente D, Murakami S, Hui R, et al Overall survival with durvalumab after chemoradiotherapy in stage III NSCLC. N Engl J Med. 2018;379(24):2342–50. 10.1056/NEJMoa1809697 30280658

[25] Coy J, Caldwell A, Chow L, Guth A, Dow S. PD-1 expression by canine T cells and functional effects of PD-1 blockade. Vet Comp Oncol. 2017 12;15(4):1487–502. 10.1111/vco.12294 28120417

[26] Maekawa N, Konnai S, Takagi S, Kagawa Y, Okagawa T, Nishimori A, et al A canine chimeric monoclonal antibody targeting PD-L1 and its clinical efficacy in canine oral malignant melanoma or undifferentiated sarcoma. Sci Rep. 2017;7(1):8951 10.1038/s41598-017-09444-2 28827658 PMC5567082

[27] Nemoto Y, Shosu K, Okuda M, Noguchi S, Mizuno T. Development and characterization of monoclonal antibodies against canine PD-1 and PD-L1. Vet Immunol Immunopathol. 2018;198:19–25. 10.1016/j.vetimm.2018.02.007 29571514

[28] Hartley G, Faulhaber E, Caldwell A, Coy J, Kurihara J, Guth A, et al Immune regulation of canine tumour and macrophage PD‐L1 expression. Vet Comp Oncol. 2017;15(2):534–49. 10.1111/vco.12197 26842912

[29] Maekawa N, Konnai S, Ikebuchi R, Okagawa T, Adachi M, Takagi S, et al Expression of PD-L1 on Canine Tumor Cells and Enhancement of IFN-γ Production from Tumor-Infiltrating Cells by PD-L1 Blockade. PLoS One. 2014;9(6). 10.1371/journal.pone.0098415 24915569 PMC4051644

[30] Tam H, Melo MBB, Kang M, Pelet JMM, Ruda VMM, Foley MHH, et al Sustained antigen availability during germinal center initiation enhances antibody responses to vaccination. Proc Natl Acad Sci. 2016;113(43). 10.1073/pnas.1606050113 27702895 PMC5086995

[31] Spanier JA, Frederick DR, Taylor JJ, Heffernan JR, Kotov DI, Martinov T, et al Efficient generation of monoclonal antibodies against peptide in the context of MHCII using magnetic enrichment. Nat Commun. 2016 9 13;7(1):11804 10.1038/ncomms11804 27292946 PMC4909947

[32] Withers SS, Moore PF, Chang H, Choi JW, McSorley SJ, Kent MS, et al Multi-color flow cytometry for evaluating age-related changes in memory lymphocyte subsets in dogs. Dev Comp Immunol. 2018;87:64–74. 10.1016/j.dci.2018.05.022 29859828 PMC6197816

[33] Kol A, Foutouhi S, Walker NJ, Kong NT, Weimer BC, Borjesson DL. Gastrointestinal Microbes Interact with Canine Adipose-Derived Mesenchymal Stem Cells In Vitro and Enhance Immunomodulatory Functions. Stem Cells Dev. 2014 8 15;23(16):1831–43. 10.1089/scd.2014.0128 24803072 PMC4120524

[34] Hewitt RE, Pele LC, Tremelling M, Metz A, Parkes M, Powell JJ. Immuno-inhibitory PD-L1 can be induced by a Peptidoglycan/NOD2 mediated pathway in primary monocytic cells and is deficient in Crohn’s patients with homozygous NOD2 mutations. Clin Immunol. 2012 5;143(2):162–9. 10.1016/j.clim.2012.01.016 22397822

[35] Kumar SR, Kim DY, Henry CJ, Bryan JN, Robinson KL, Eaton AM. Programmed death ligand 1 is expressed in canine B cell lymphoma and downregulated by MEK inhibitors. Vet Comp Oncol. 2017; 10.1111/vco.12297 28111882

[36] Wang C, Thudium KB, Han M, Wang X-T, Huang H, Feingersh D, et al In Vitro Characterization of the Anti-PD-1 Antibody Nivolumab, BMS-936558, and In Vivo Toxicology in Non-Human Primates. Cancer Immunol Res. 2014 9 1;2(9):846–56. 10.1158/2326-6066.CIR-14-0040 24872026

[37] Panjwani MK, Smith JB, Schutsky K, Gnanandarajah J, O’Connor CM, Powell DJ, et al Feasibility and safety of RNA-transfected CD20-specific chimeric antigen receptor T cells in dogs with spontaneous B cell lymphoma. Mol Ther. 2016;24(9):1602–14. 10.1038/mt.2016.146 27401141 PMC5113111

[38] Maekawa N, Konnai S, Okagawa T, Nishimori A, Ikebuchi R, Izumi Y, et al Immunohistochemical analysis of PD-L1 expression in canine malignant cancers and PD-1 expression on lymphocytes in canine oral melanoma. PLoS One. 2016;11(6). 10.1371/journal.pone.0157176 27276060 PMC4898770

[39] Canter RJ, Grossenbacher SK, Foltz JA, Sturgill IR, Park JS, Luna JI, et al Radiotherapy enhances natural killer cell cytotoxicity and localization in pre-clinical canine sarcomas and first-in-dog clinical trial. J Immunother Cancer. 2017 12 19;5(1):98 10.1186/s40425-017-0305-7 29254507 PMC5735903

[40] Gibney GT, Weiner LM, Atkins MB. Predictive biomarkers for checkpoint inhibitor-based immunotherapy. Lancet Oncol. 2016 12 1;17(12):e542–51. 10.1016/S1470-2045(16)30406-5 27924752 PMC5702534

[41] Lee HH, Wang YN, Xia W, Chen CH, Rau KM, Ye L, et al Removal of N-Linked Glycosylation Enhances PD-L1 Detection and Predicts Anti-PD-1/PD-L1 Therapeutic Efficacy. Cancer Cell. 2019 8 12;36(2):168-178.e4. 10.1016/j.ccell.2019.06.008 PMC6793936 31327656

[42] Topalian SL, Taube JM, Anders RA, Pardoll DM. Mechanism-driven biomarkers to guide immune checkpoint blockade in cancer therapy. Nat Rev Cancer. 2016 5 15;16(5):275–87. 10.1038/nrc.2016.36 27079802 PMC5381938

[43] Bu X, Mahoney KM, Freeman GJ. Learning from PD-1 Resistance: New Combination Strategies. Trends Mol Med. 2016 6;22(6):448–51. 10.1016/j.molmed.2016.04.008 27174038 PMC6833952

[44] Mantovani A, Marchesi F, Malesci A, Laghi L, Allavena P. Tumour-associated macrophages as treatment targets in oncology. Nat Rev Clin Oncol. 2017 7 24;14(7):399–416. 10.1038/nrclinonc.2016.217 28117416 PMC5480600

[45] Thommen DS, Schumacher TN. T Cell Dysfunction in Cancer. Cancer Cell. 2018 4;33(4):547–62. 10.1016/j.ccell.2018.03.012 29634943 PMC7116508

