# Supplementary material for: Development of canine PD-1/PD-L1 specific monoclonal antibodies and amplification of canine T cell function
Source: PLoS One. 2020 Jul 2;15(7):e0235518. doi: 10.1371/journal.pone.0235518 (PMC7332054; doi:10.1371/journal.pone.0235518)
Supplement: S1 Table — (DOCX) [file pone.0235518.s001.docx]

| Dogs | Species | Age | Related figure |
| --- | --- | --- | --- |
| Dog 1 | Labrador Boxer mix (MC) | 4 | Figure 5 |
| Dog 2 | Husky mix (FS) | 12 | Figure 5 |
| Dog 3 | Doberman pincher (MC) | 1.5 | Figure 6 |
| Dog 4 | Golden retriever (MC) | 3 | Figure 6 |
| Dog 5 | German Shephard mix (FS) | 4 | Figure6 |
| Dog 6 | English bull dog mix (MC) | 7 | Figure 6 |
| Dog 7 | German Shephard (MC) | 5 | Figure 8 |

FS: represents female spayed. MC: represent male castrated
