# Supplementary material for: Development of canine PD-1/PD-L1 specific monoclonal antibodies and amplification of canine T cell function
Source: PLoS One. 2020 Jul 2;15(7):e0235518. doi: 10.1371/journal.pone.0235518 (PMC7332054; doi:10.1371/journal.pone.0235518)
Supplement: S2 Table — (DOCX) [file pone.0235518.s002.docx]

| Target/Species | Conjugates | Host/Isotype | Clone # | Manufacturer | Catalogue # | Dilution |
| --- | --- | --- | --- | --- | --- | --- |
| IgA/Mouse | Biotin | Rat/IgG1,k | 11-44-2 (Monoclonal) | Thermo Fisher Scientific | 13-5994-82 | 1:100 |
| IgG/Human | PE | Mouse/IgG2a,k | HP6017 | Biolegend | 409303 | 1:100 |
| IgG/Mouse | HRP | Goat/IgG | Polyclonal | Biolegend | 405306 | 1:2000 |
| IgG/Mouse | FITC | Goat/Ig, F(ab’)2 | Polyclonal | Thermo Fisher Scientific | 11401082 | 1:100 |
| CD4/Dog | PacBlule | Rat/IgG2a | YKIX302.9 (Monoclonal) | Bio-Rad | MCA1038PB | 1:10 |
| CD8a/Dog | PerCP-EF710 | Rat/IgG1,k | YCATE55.9 (Monoclonal) | Thermo Fisher Scientific | 46-5080-42 | 1:25 |
| CD3/Human | FITC | Rat/IgG1 | CD3-12 (Monoclonal) | Bio-Rad | MCA1477FT | 1:50 |
| CD5/Dog | PerCP-EF710 | Rat/IgG2a,k | YKIX322.3 | Thermo Fisher Scientific | 46-5050-42 | 1:10 |
| MHCII/Dog | FITC | Rat/IgG2a | YKIX334.2 | Bio-Rad | MCA1044F | 1:10 |
| CD14/Human | Qdot605 | Mouse/IgG2a | Tuk4 | Thermo Fisher Scientific | Q10013 | 1:25 |
| CD11c/Dog | PE | Mouse/IgG1 | CA11.6A1 | Peter Moore Lab | N/A | 1:50 |
| PD-1/Dog | None/PE/APC | Mouse/IgA | JC053 | Current study | N/A | 30 ug/ml |
| PD-L1/Dog | None/PE/APC | Mouse/IgG1 | JC071 | Current study | N/A | 10-30 ug/ml |
| PD-L1/Dog | None/PE/APC | Mouse/IgG2a | JC173 | Current study | N/A | 30 ug/ml |
| PD-L1/Dog | None/PE/APC | Mouse/IgG1 | JC194 | Current study | N/A | 30 ug/ml |
| PD-L1/Dog | None/PE/APC | Mouse/IgG1 | JC205 | Current study | N/A | 30 ug/ml |
| Isotype control | N/A | Mouse/IgG1,k | 11711 | R&D Systems | MAB002 | Matched |
| Isotype control | N/A | Mouse/IgG2a,k | G155-788 | BD Bioscience | 555571 | Matched |
| Isotype control | N/A | Mouse/IgA | S107 | Thermo Fisher Scientific | 11-47662-81 | Matched |
| Streptavidin | AP | N/A | N/A | Thermo Fisher Scientific | 434322 | 1:1000 |
| Streptavidin | HRP | N/A | N/A | Biolegend | 405210 | 1:1000 |
| Streptavidin | RPE | N/A | N/A | Agilent | PJRS25 | 1:8.2 |
| Streptavidin | FITC | N/A | N/A | BD Bioscience | 11-4311-87 | 1:100 |
| Streptavidin | APC | N/A | N/A | Thermo Fisher Scientific | 17-4317-82 | 1:100 |
