# Supplementary figures and images for: Development of canine PD-1/PD-L1 specific monoclonal antibodies and amplification of canine T cell function
Source: PLoS One. 2020 Jul 2;15(7):e0235518. doi: 10.1371/journal.pone.0235518 (PMC7332054; doi:10.1371/journal.pone.0235518)

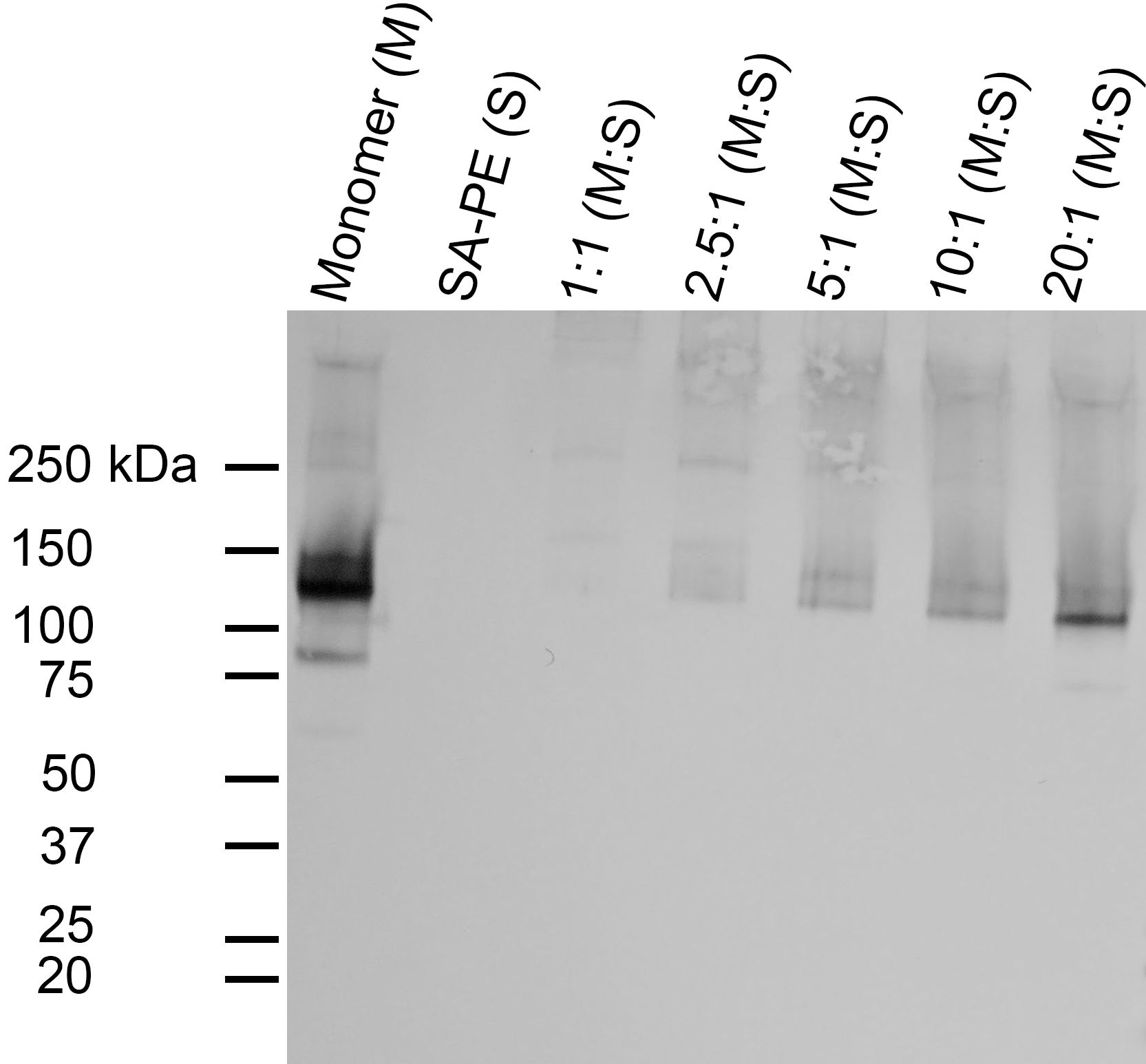

Supplement: S1 Fig — Each monomer was mixed with SA-PE to generate tetramer. To find correct ratio between PD-1Ig and SA-PE for tetramer generation, different ratios of monomer and SA-PE were tested on Western blot in non-reducing condition. Samples are monomer only (lane 1), SA-PE only (lane 2), monomer and SA-PE with 1:1 (lane 3), 2.5:1 (lane 4), 5:1 (lane 5), 10:1 (lane 6), and 20:1 molar ratio (lane 7). Correct ratio was determined to be 2.5:1 ratio for both PD-1Ig. (TIF) [file pone.0235518.s003.tif]

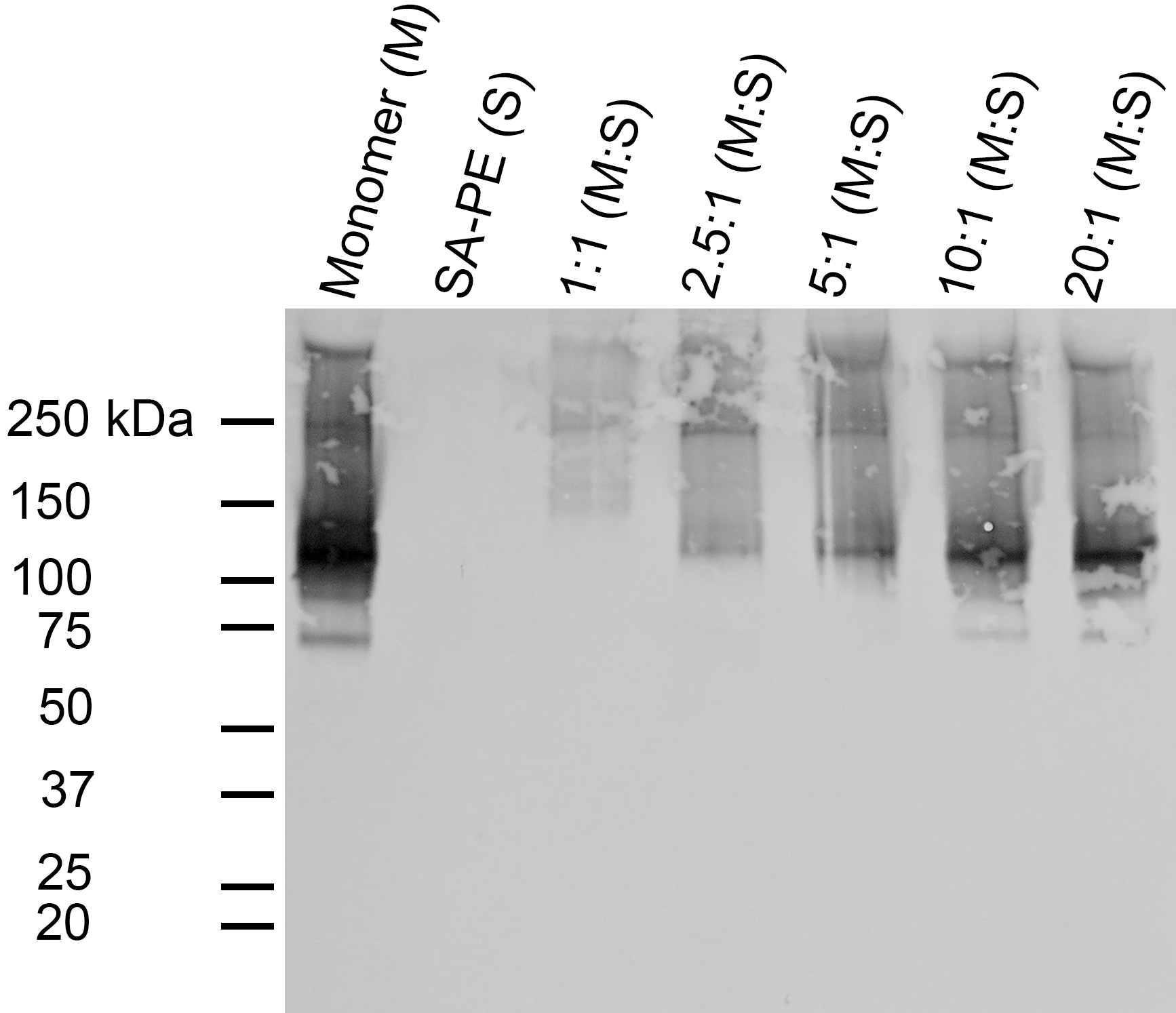

Supplement: S2 Fig — Each monomer was mixed with SA-PE to generate tetramer. To find correct ratio between PD-L1Ig and SA-PE for tetramer generation, different ratios of monomer and SA-PE were tested on Western blot in non-reducing condition. Samples are monomer only (lane 1), SA-PE only (lane 2), monomer and SA-PE with 1:1 (lane 3), 2.5:1 (lane 4), 5:1 (lane 5), 10:1 (lane 6), and 20:1 molar ratio (lane 7). Correct ratio was determined to be 2.5:1 ratio for both PD-L1Ig. (TIF) [file pone.0235518.s004.tif]

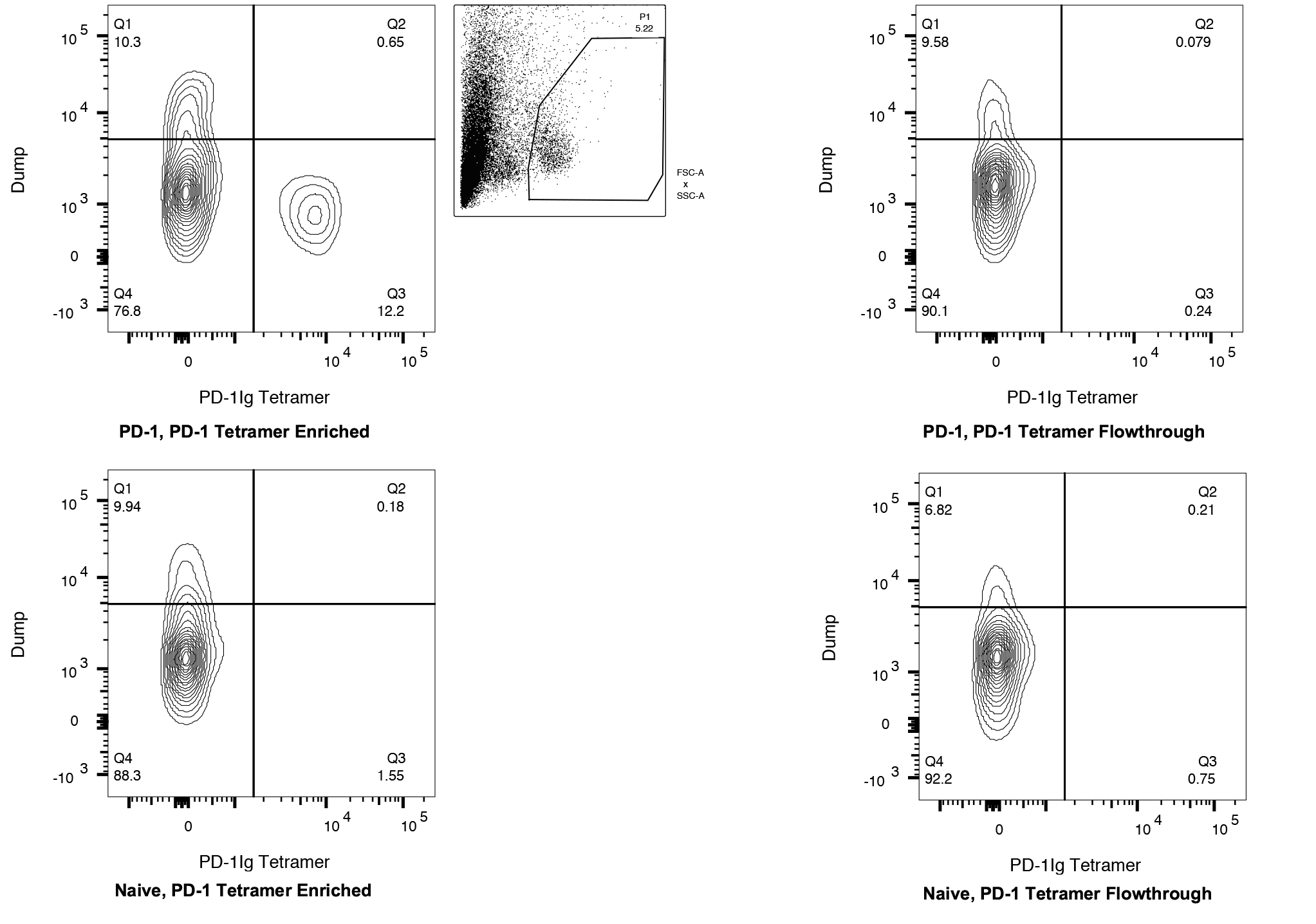

Supplement: S3 Fig — Dump- Tetramer+ frequency for PD-1Ig immunized sample can be compared to naïve sample when PD-1Ig tetramer was applied. (TIF) [file pone.0235518.s005.tif]

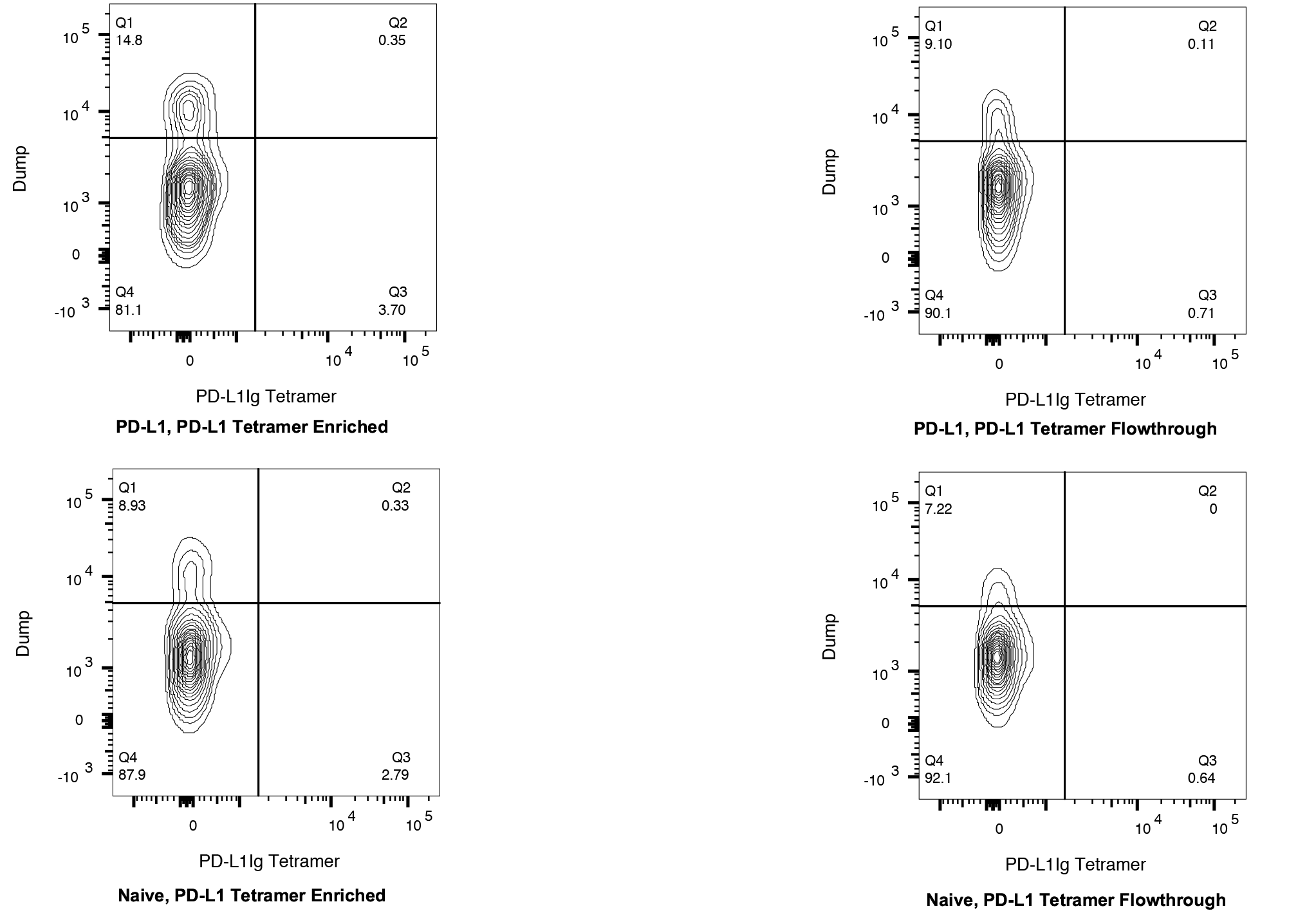

Supplement: S4 Fig — Dump Tetramer+ frequency for PD-L1Ig immunized sample can be compared to naïve sample when PD-L1Ig tetramer was applied. (TIF) [file pone.0235518.s006.tif]

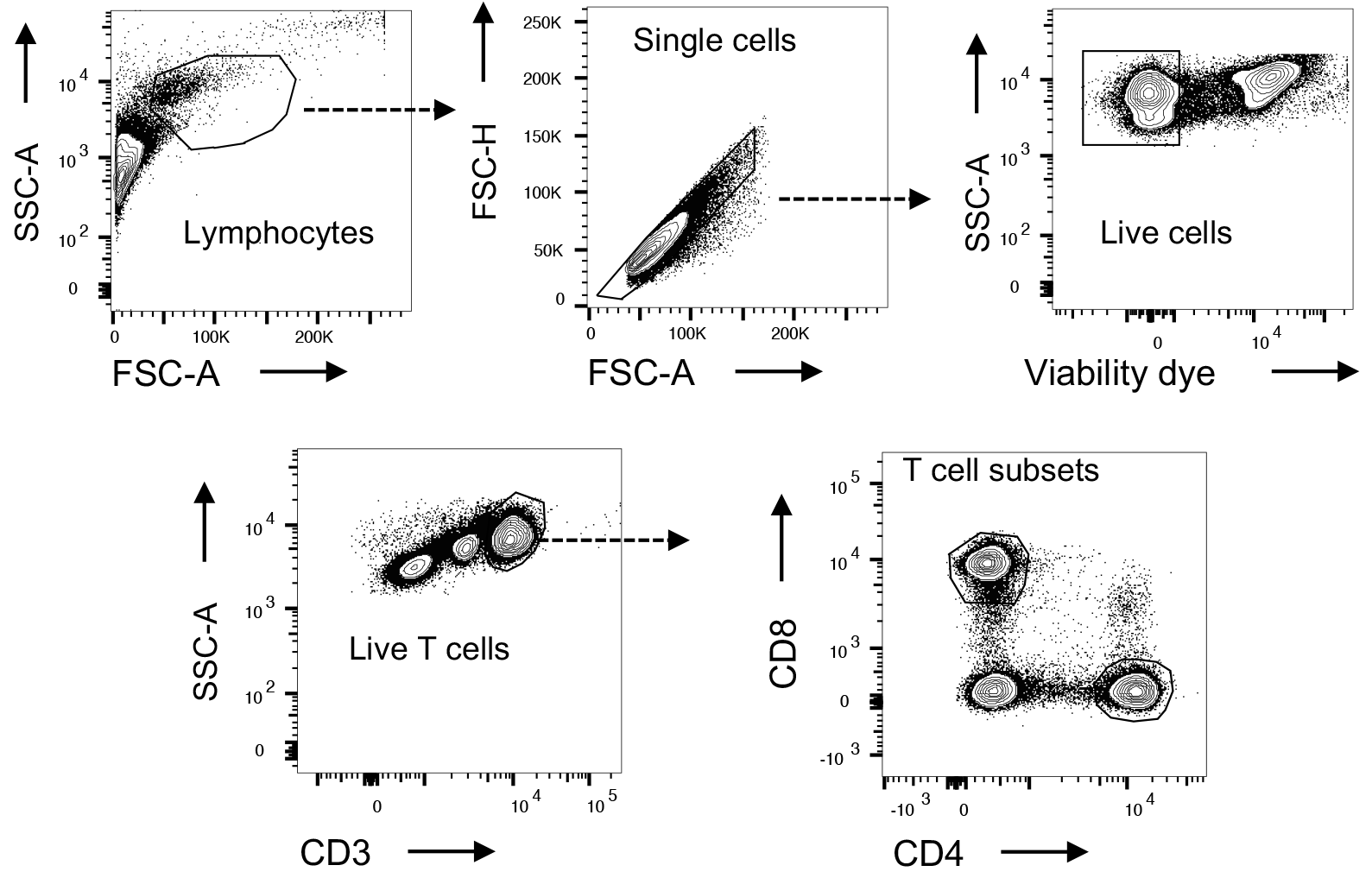

Supplement: S5 Fig — A standard gating strategy used for CD4+ and CD8+ T cell subsets by flow cytometry and for analysis of frequencies of PD-1+ populations is shown. (TIF) [file pone.0235518.s007.tif]

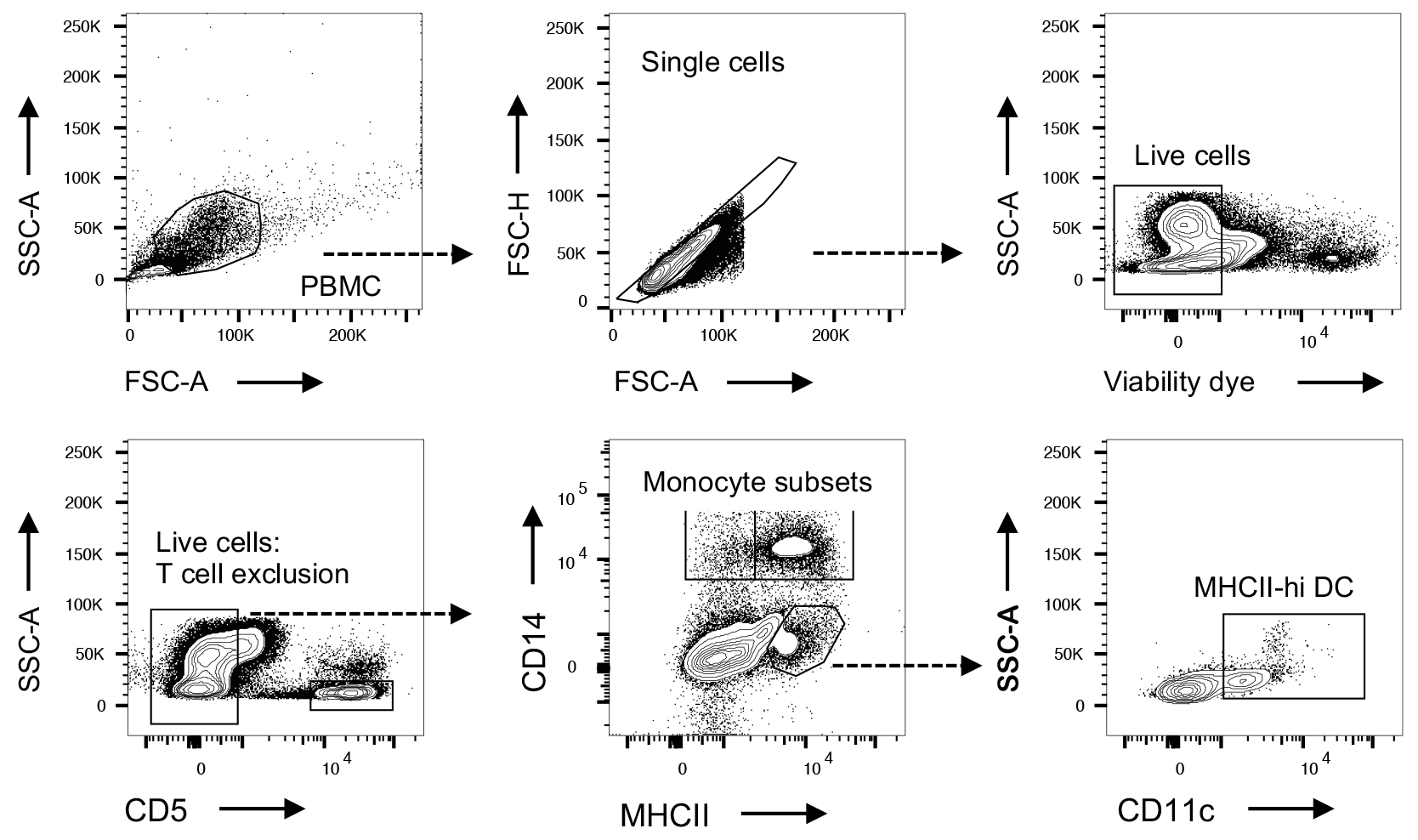

Supplement: S6 Fig — The basic gating strategy used for immune cell subsets by flow cytometry and for analysis of frequencies of PD-L1+ populations is shown. Subsets of interest included CD5-MHCII+CD14+ and CD5-MHCII-CD14+ monocytes and DC defined as CD5-MHCIIhiCD14-CD11c+. (TIF) [file pone.0235518.s008.tif]

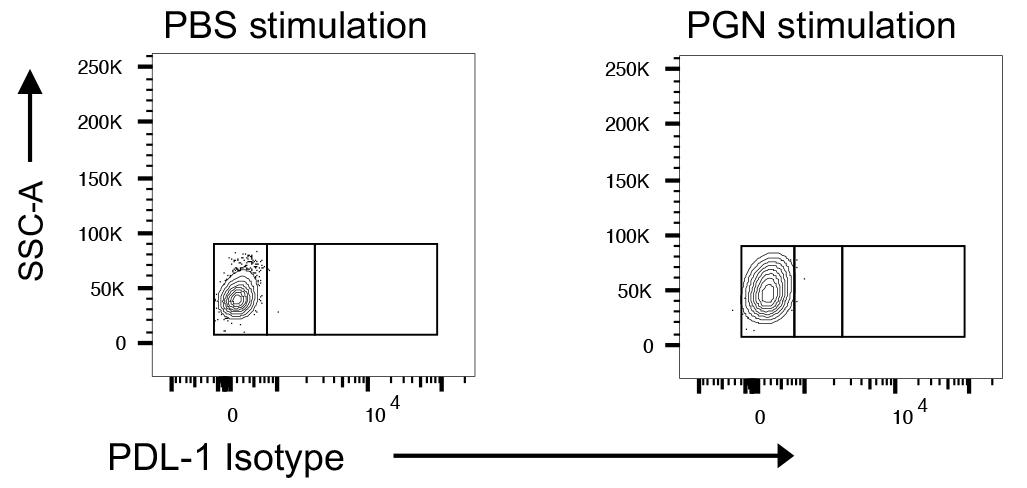

Supplement: S7 Fig — Staining of the CD5-MHCII+CD14+ subset before and after PGN stimulation with an isotype control antibody is also shown. (TIF) [file pone.0235518.s009.tif]

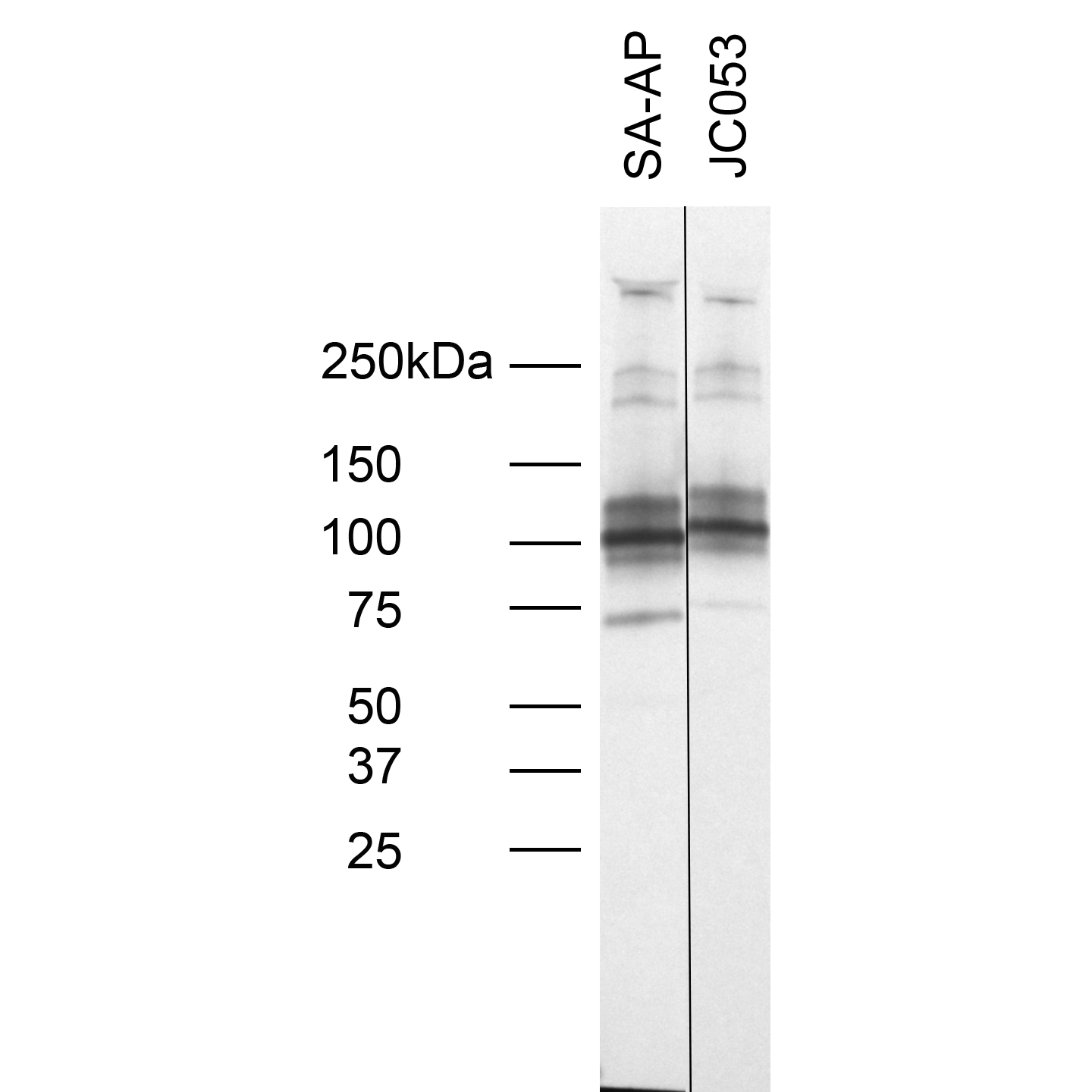

Supplement: S8 Fig — Soluble PD-1Ig was detected on Western blot in non-reducing condition using JC053, and anti-mouse IgG-AP, sequentially (Right). This was compared to biotinylated PD-1Ig detected using SA-AP (Left). Two blots using SA-AP and JC053 were prepared on separate membranes. (TIF) [file pone.0235518.s010.tif]

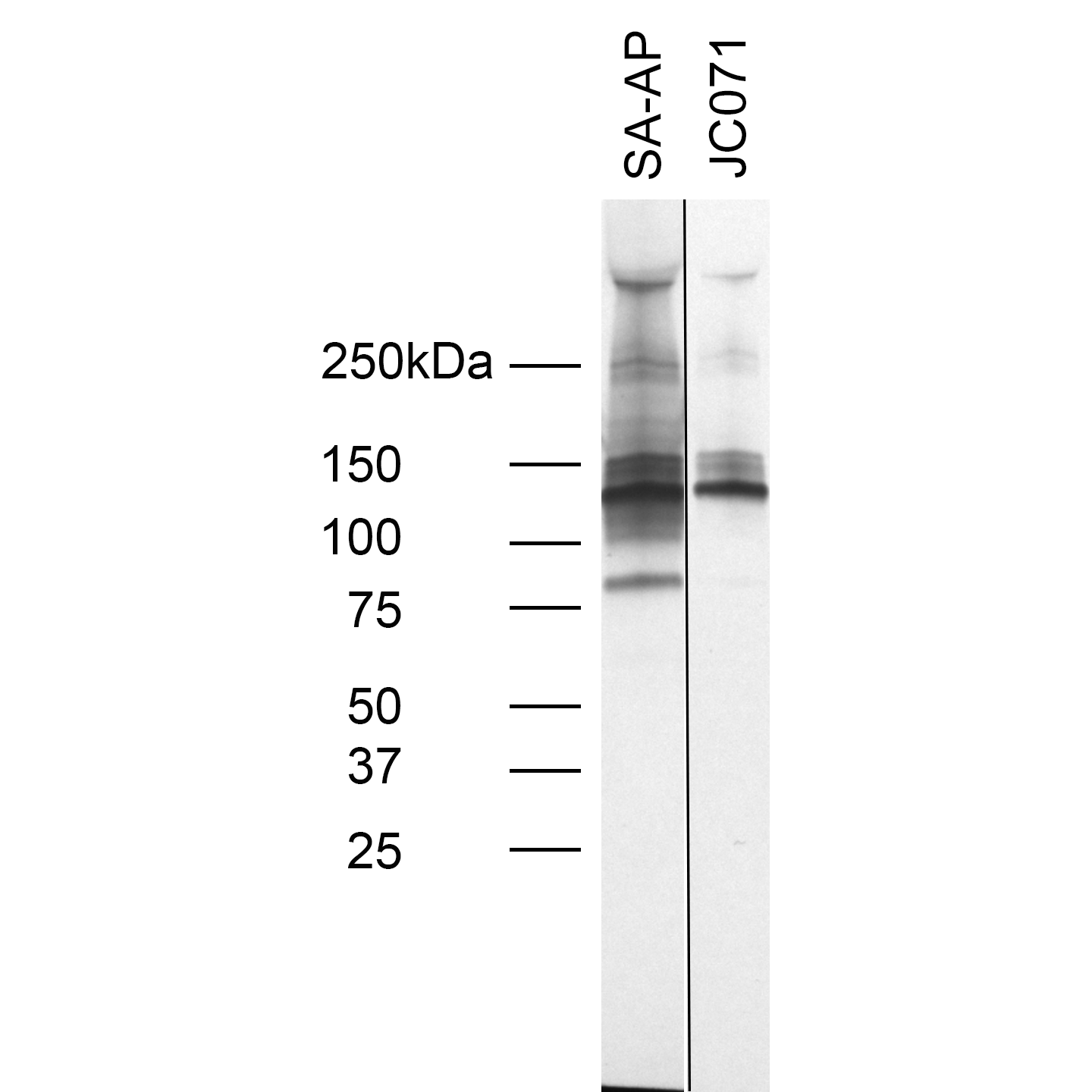

Supplement: S9 Fig — Soluble PD-L1Ig expressed in S2 was detected on Western blot in non-reducing condition using JC071 and anti-mouse IgG-AP, sequentially (Right). This was again compared to SA-AP treated blot (Left). Two blots using SA-AP and JC071 were prepared on separate membranes. (TIF) [file pone.0235518.s011.tif]

S1 Fig Original

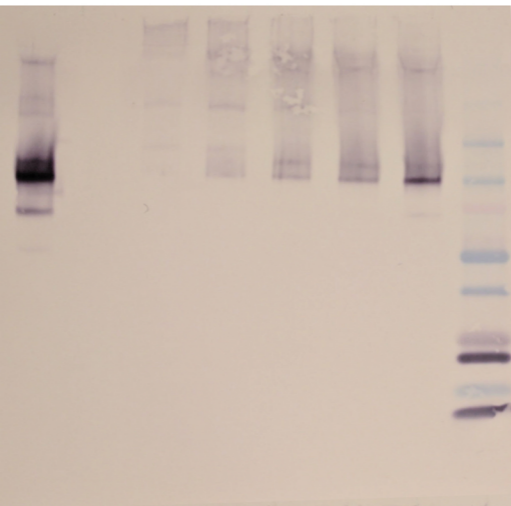

S2 Fig Original

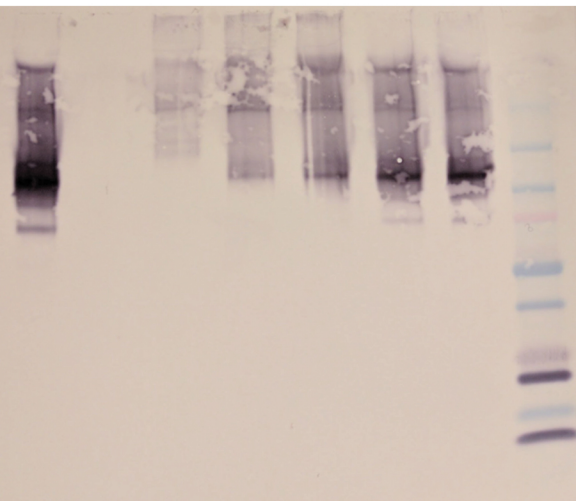

# S8 Fig JC053 Original

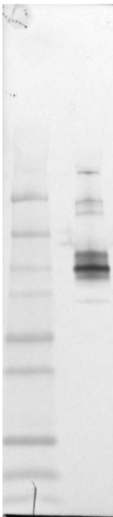

# S9 Fig JC071 Original

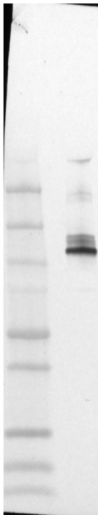

S8-9 Fig SA-AP Original

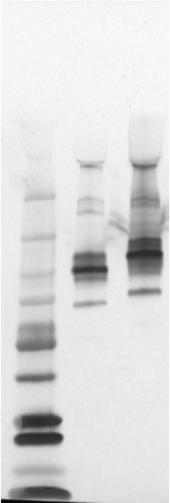

Supplement: S1 Raw images — (PDF) [file pone.0235518.s012.pdf]
